# Characterizing criticality of proteins by systems dynamics: *Escherichia coli *central carbon metabolism as a working example

**DOI:** 10.1186/1752-0509-6-S1-S11

**Published:** 2012-07-16

**Authors:** Ru-Dong Li, Lei Liu

**Affiliations:** 1 Key Laboratory of Systems Biology, Shanghai Institutes for Biological Sciences, Chinese Academy of Sciences, Shanghai, China; 2 Shanghai Center for Bioinformatics Technology, Shanghai, China

## Abstract

**Background:**

Systems biology calls for studying system-level properties of genes and proteins rather than their individual chemical/biological properties, regarding the bio-molecules as system components. By characterizing how critical the components are to the system and classifying them accordingly, we can study the underlying complex mechanisms, facilitating researches in drug target selection, metabolic engineering, complex disease, etc. Up to date, most studies aiming at this goal are confined to the topology-based or flux-analysis approaches. However, proteins have tertiary structures and specific functions, especially in metabolic systems. Thus topological properties such as connectivity, path length, etc., are not good surrogates for protein properties. Also, the manner of individual sensitivity analysis in most flux-analysis approaches cannot reveal the simultaneous impacts on collateral components as well as the overall impact on the system, thus lacking in system-level perspective.

**Results:**

In the present work, we developed a method to directly assess protein system-level properties based on system dynamics and *in silico *knockouts, regarding to the conceptual term "criticality". Applying the method to *E. coli *central carbon metabolic system, we found that multiple enzymes including phosphoglycerate kinase, enolase, transketolase-b, etc., had critical roles in the system in terms of both system states and dynamical stability. In contrast, another set of enzymes including glucose-6-phosphate isomerise, pyruvate kinase, phosphoglucomutase, etc., exerted very little influences when deleted. The finding is consistent with experimental characterization of metabolic essentiality and other studies on *E. coli *gene essentiality and functions. We also found that enzymes could affect distant metabolites or enzymes even greater than a close neighbour and asymmetry in system-level properties of enzymes catalyzing alternative pathways could give rise to local flux compensation.

**Conclusions:**

Our method creates a different angle for evaluating protein criticality to a biological system from the conventional methodologies. Moreover, the method leads to consistent results with experimental references, showing its efficiency in studying protein system-level properties. Besides working on metabolic systems, the application of the method can be extended to other kinds of bio-systems to reveal the constitutive/functional properties of system building blocks.

## Background

Systems biology focuses on studying properties of bio-molecules like genes and proteins at the system level, especially their constitutive/functional roles as system components. By exploring their interplay structure in the system, we can evaluate how critical a building block is and how different parts vary in properties [1,2]. Based on such knowledge, we can understand how a system is formed, how the system-level function is achieved and whether it can be modified according to our needs, enhancing researches in drug target selection, metabolic engineering, complex diseases, etc [3,4]. *E. coli *is the best-studied organism, with knowledge accumulated in each of its biological hierarchies, e.g. genetic regulation, genomic information, metabolism, etc [5-7]. The central carbon metabolism contains glycolysis and pentose phosphate pathways as principal parts (Additional file 1). It is the most common and conservative pathway among prokaryotes, with close resemblance in eukaryotes [5,7,8].

Up to date, multiple genome-scale networks have been built on the organism with regard to the pathway to reveal essentiality of genes and proteins [9,10]. However, most of such studies are based on network topology or flux analysis. In topology-based approaches, system-level properties are defined as the connectivity of a molecule or shortest path lengths, etc [11,12]. Such properties usually have poor consistency with experimental characterizations, especially on the protein level. For example, multiple studies suggest that proteins with large connectivity in protein-protein interaction networks are not essential. Also, many enzymes associated with large number of accompanies exert very little influence on cell growth [6,13,14]. We think the possible reason is that mere topology does not encode specific biochemical/biological functions of proteins, whereas topology-based approaches purely regard the bio-molecules as vertices in an abstract graph. While in flux-analysis approaches (e.g. flux balance analysis - FBA; metabolic control analysis - MCA), they calculate the extent of how a perturbation on a system parameter influences a specific, pre-defined system objective. Although such individual sensitivity analyses give a quantitative measure of a component's control on a functional pathway, they cannot reveal the simultaneous impacts on other parts of the system and the overall system. In other words, besides the pre-defined objective in interest, we will not know if a perturbation triggers collateral influences on other parts of the system and what it implicates to the overall system. The lack in system-level perspective possibly gives rise to false positive results, because a simulated perturbation favouring an objective may not be actually carried out as we do not know if it has lethal impacts on collateral but crucial components in the system.

**Figure 1:**
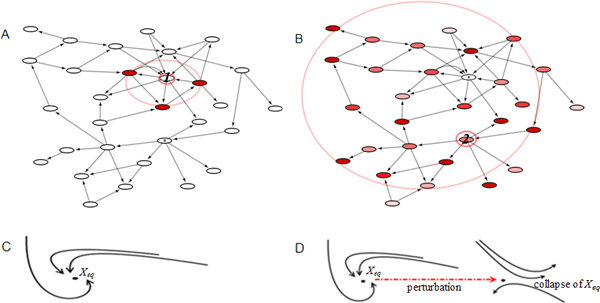
**Schematic illustration of the protein criticality characterization method**. System components may exert different impacts when perturbed. Color gradient corresponds to amplitudes, with empty color representing zero effect. (A) Circumstance that the impact is within a limited area near the epicenter (i.e. the perturbed component, marked as "1"). (B) Circumstance that the impact spreads throughout the network, with many distant spots severely affected (marked as "2"). (C) A stable equilibrium (*X_eq_*) attracts its neighboring trajectories. (D) An unstable equilibrium repels the trajectories. Stable equilibriums may collapse due to perturbations on critical system components. Components whose perturbations exert large impacts, or cause qualitative changes in system dynamics (upon perturbation) are regarded as critical.

Under such consideration, we developed a new method to characterize protein criticality based on kinetic systems, which can accurately reflect system behaviours and has explicit context on the biophysical/biochemical basis [5,15]. Because *E. coli *central carbon metabolism is the only system with comprehensive kinetic data, we used it as our model. The system components (bio-molecules) were the enzymes, and we defined the criticality of a component by its *in silico *knockout. We explored how the deletion of an enzyme influenced the system state, i.e. whether state fluctuations were restricted in a limited area or spread throughout a broader range; and how large their amplitudes were. Moreover, we investigated the dynamical stability of the residual system to see whether the system maintained or lost metabolic robustness after removing the enzyme (Figure 1). From these computations, we characterized the criticality of proteins and our results were consistent with published experiments. Furthermore, our method may create a new viewpoint for protein system-level property characterization, which differs from conventional methodologies and is more comprehensive for analyzing complex systems.

## Results

We computed the criticality for all enzymes in the system, and observed that they can be categorized into two classes: those with critical properties and those with uncritical properties. An enzyme is characterized as critical if its deletion caused large influences on system states and qualitative changes to system dynamical stability.

### System state fluctuation

We first simulated the system to obtain metabolite kinetics and flux distributions under normal conditions. Next, we carried out *in silico *enzyme knockouts by modifying the corresponding parameters and re-simulating the system. Following the definition in previous studies, we regarded concentrations as the primary system state [6,16]. We calculated state deviations of the modified system and computed the fluctuation amplitude of each metabolite upon the enzyme's removal. Here we encoded them with a vector *f*. Second, we assessed the impact area by calculating the distances of metabolites from the removed enzyme and encoded them with a vector *d*. This allowed us to see whether the influence was within a limited radius or propagated to distant parts of the system. In short, we used a vector pair *U = (d,f) *to represent system state fluctuation, and we could quantify the overall impact with a measurement formula (see section "Methods" for details). All results here were summarized in Additional file 2.

**Figure 2:**
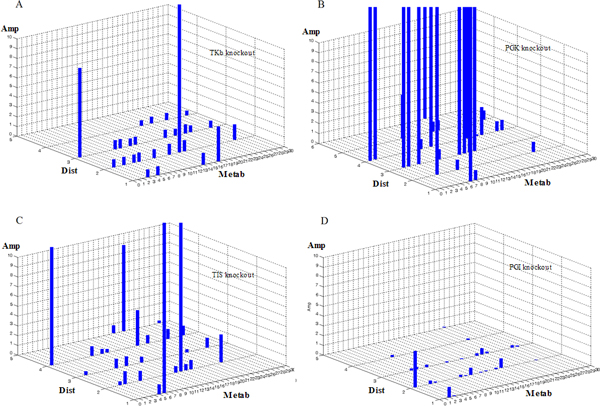
**System state fluctuations caused by enzyme deletions**. The impacts of enzyme deletions on system states are shown. In all subfigures, x-axis: state variable (metabolite) indexes (denoted as "Metab", see Additional file 6 for details); y-axis: distance of state variable from the deleted enzyme ("Dist"); z-axis: the impact amplitude ("Amp"). (A - D) The influences of TKb, PGK, TIS, and PGI deletions on system state, respectively. The figures exemplify that an enzyme can affect distant metabolites even greater than its closest neighbours.

We discovered that many enzymes could exert (upon deletion) large influences on the kinetics of many metabolites, i.e. caused large system state fluctuations if deleted. For example, transketolase-b (TKb), an enzyme catalyzing a coupling branch of the glycolysis and the pentose phosphate pathways, had relatively large influences on many metabolites in the central carbon metabolism, especially for glucose, sedoheptulose-7-phosphate, and erythrose-4-phosphate (Figure 2A). Phosphoglycerate kinase (PGK), the enzyme catalyzing the conversion between 1,3-diphosphoglycerate and 3-phosphoglycerate on a linear branch in the glycolysis pathway, exerted even greater impacts on these metabolites as well as other ones throughout the system such as glucose-6-phosphate, fructose-6-phosphate, glyceraldehydes-3-phosphate, ribulose-5-phosphate, etc. Moreover, it could also exert large impact on oxaloacetate, an intermediate in the tricarboxylic acid (TCA) cycle, as well as on polysaccharide synthesis, an external pathway connected with central carbon metabolism (Figure 2B). Similarly, enzymes at other locations such as enolase (ENO), glyceraldehydes-3-phosphate dehydrogenase (GAPDH), ribose-5-phosphate isomerase (R5PI), aldolase (ALDO), transaldolase (TA), etc., also exhibited large impacts on system states (Additional file 2). The overall influences of PGK, ENO, and GAPDH were superior to those of ALDO, R5PI, TKb and TA, especially at the distance ≥ 4 and 5 (Figure 2, Additional file 2). This indicated that enzymes like PGK, ENO and GAPDH could impact distant areas more strongly and exert a more persistent impact with respect to system structure. Noteworthy, triosephosphate isomerase (TIS), which catalyzed inter-conversions between glyceraldehydes-3-phosphate and its isomer dihydroxyacetonephosphate, would be regarded as a peripheral component in the system by traditional topology-based and flux-analysis approaches as it was not on any uni-directional or rate limiting steps. However, our computation results showed that its deletion also resulted in large impact (Figure 2C). The difference in prediction was because our method assumed that the influence exerted by an enzyme was not only depend its location (network topology), but also determined by its parametric properties (kinetic parameters).

Meanwhile, we also found that there were another group of enzymes, in contrast to those mentioned above, having very little influences on system states. For example, multiple enzymes such as glucose-6-phosphate isomerase (PGI), pyruvate kinase (PK), phosphoglucomutase (PGM), etc., only resulted in very small state fluctuations when deleted. The amplitudes were slight, and the influences were mostly within limited areas as the amplitudes were negligible at the distance ≥ 2 (Figure 2D, Additional file 2). Hence, for system-level properties so far as system state fluctuation was considered, the former enzymes were much greater to those in the latter group.

More interestingly, we found that the most severely influenced metabolites did not always concentrate in the close neighborhood of the perturbed enzyme. For example, the largest impacts of TKb deletion were at the distances of 2 and 3 but not at the distance of 1 (Figure 2A). Likewise, the largest impacts of PGK deletion occurred at the distances of 3 and 4 also not at the distance of 1 (Figure 2B). Similar patterns were also seen from the results of other enzymes like ENO, R5PI, ALDO, GAPDH, TA and PGI (Figure 2, Additional file 2). This suggested that in contrast to the intuition that perturbation would cause largest changes to its neighborhood, distant effects could occur due to the leverage of system dynamics.

We also examined the impacts of enzyme knockouts from the enzyme-centric view with our method. With each enzyme representing a reaction and using fluxes as system states, we computed flux change amplitudes and impact radiuses on the enzyme-centric network in the same way stated previously. The results showed a similar pattern with the results presented here (Additional file 3).

### System dynamical stability

**Figure 3:**
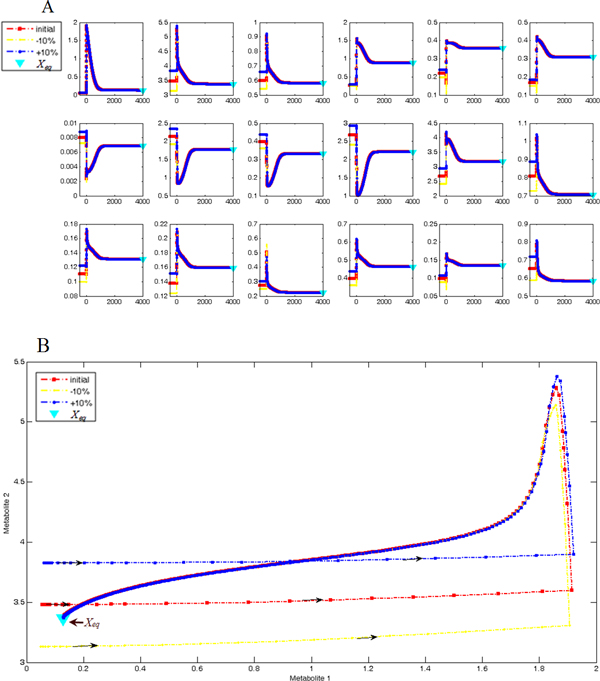
**The asymptotically stable equilibrium point**. The stable equilibrium (*X_eq_*) is illustrated by trajectories and phase orbits in the 18D space with 10% perturbations. The red line: the original curve with respect to the experimental initial value [5]; the yellow/blue lines: the curves whose initial value has a -10%/+10% perturbation from the experimental one; the triangular spots: projections of *X_eq _*on the corresponding dimensions. (A) Asymptotical stability shown by trajectories. Each subplot represents a dimension in the 18D space, i.e. the kinetics of a metabolite. All trajectories eventually and consistently converge to the *X_eq _*(projection on the corresponding dimension) although a 10% perturbation is in the initial value. The x-axis: time (s); y-axis: concentration (mM). (B) Asymptotical stability shown by phase orbits. Stability is more clearly illustrated in such presentation. We randomly chose 2 state variables (metabolites #7 and #8 in the plot) to form the phase orbit in the 2D subspace. Arrows denote the directions of orbits and they eventually and consistently converge to *X_eq _*(projection on the 2D subspace, marked by the triangular spot). For other 2D subspaces, the orbit profiles are the same.

We found that the original system had an asymptotically stable equilibrium point *X_eq _*in a large range of ordinary intracellular concentrations in the parameter/state space, which made all trajectories in a wide neighborhood tending to it (Figure 3). This gives rise to metabolic robustness, as slight perturbations in initial values do not cause large changes in system states [17,18]. As it is well in accord with the Lyapunov stability, we could characterize an enzyme's criticality by examining the bifurcations of *X_eq _*with respect to the deletion of the enzyme. Such bifurcations included: (1) whether deleting this enzyme made the residual system have no equilibrium; (2) if the residual system still had equilibrium(s), how far its location deviated from *X_eq_*; and (3) whether its stability property changed (i.e. if there are changes in the neighborhood orbit structure). Equilibrium(s) was computed by dynamical simulation and optimization methods. When it was located, its deviation from *X_eq _*was calculated and its neighborhood orbit structure was described by the rules of topological conjugacy (see the "Methods" section for details). As multiple enzyme deletions might generate topologically identical orbit structures, we showed several typical cases as examples here. See Additional file 4 for a complete catalogue of all results.

**Figure 4:**
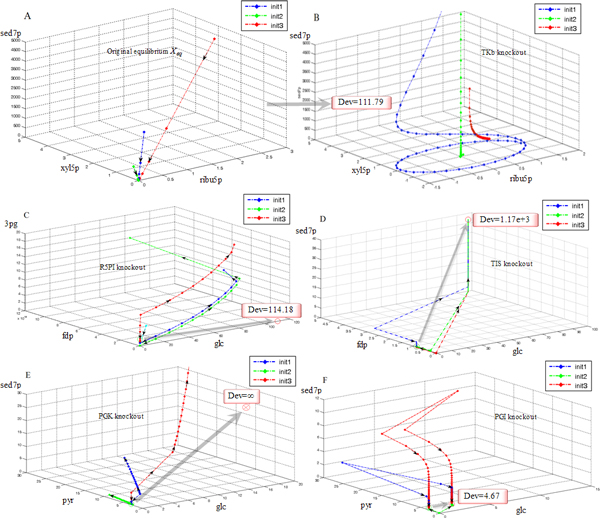
**Equilibrium deviations and orbit structure changes caused by enzyme deletions**. Impacts of enzyme deletions on dynamical stability are shown in terms of equilibrium deviations and orbit structure changes. The curves are drawn under the principle of topology conjugacy. They show the qualitative dynamics but they are not real-value trajectories. Lines in different colors represent different curves initiated at different values (init 1-3). In each subfigure, the 3 dimensions represent the metabolites that differ most from the original kinetics. (A) The original equilibrium (*X_eq_*), denoted by (0,0,0). All trajectories converge to it. (B) The unstable equilibrium after deleting TKb. The dimension *sed7p *is divergent, (*ribu5p, xyl5p*) form an unstable limit cycle. The 3 colored orbits initiated from different values lead to convergence, limit cycle and divergence on the 2D plane (*ribu5p, xyl5p*). (A) and (B) are separately drawn in order to show the limit cycle clearly. Orbits are centred at (0,0,0) to achieve a better visual effect. (C) Deleting R5PI causes a long-distance equilibrium deviation and alters the system stability. The source and target ends of the grey arrow mark *X_eq _*and the re-established equilibrium (*X_d_*) respectively, with the distance (in the metric unit of the state space) marked in the box. Initiated from identical values, *X_eq _*attracts the orbits and *X_d _*repels the orbits along the dimensions *fdp *and *3pg*. (D) Deleting TIS causes the equilibrium to deviate an even larger distance. Legends are the same. (E) Deleting PGK makes the system have no equilibrium. The deviation is denoted as infinite (∞). (F) Deleting PGI does not cause obvious changes in system dynamics. The re-established equilibrium is also asymptotically stable and it is very near to *X_eq_*. See Additional file 6 for abbreviations of metabolite names.

After *in silico *knockout of TKb, the residual system had large qualitative changes in system dynamics. It exhibited equilibrium far away from *X_eq _*with very different stability property (Figure 4A-4B). It was an unstable equilibrium with the trajectory representing sedoheptulose-7-phosphate kinetics being divergent and the two dimensions representing ribulose-5-phosphate and xylulose-5-phosphate forming a limit cycle when certain initial values held. By setting different initial values on the 2-dimensional plane of the limit cycle and investigating the trajectory dynamics, it was seen that the limit cycle was an unstable one. Trajectories on the plane inside its range converged to the equilibrium's projection on the plane; and trajectories outside its range spread quickly through both dimensions (Figure 4B). Likewise, deleting TA caused the system equilibrium to relocate to a similar distance and it had similar properties to those in the case of TKb. It was also an unstable one with one dimension being divergent and another two dimensions forming an unstable limit cycle. What differs from TKb is only that the divergent dimension was 6-phosphogluconate and the two cycling dimensions were xylulose-5-phosphate and sedoheptulose-7-phosphate. R5PI knockout also made the equilibrium shift a long distance and reversed its stability. For the neighborhood orbits, the divergent dimensions were fructose-1,6-biphosphate and 3-phosphoglycerate (Figure 4C).

Deleting TIS or ALDO caused the system to re-establish equilibrium over an extreme distance beyond the ordinary range (Figure 4D). This indicated that after such a deletion, if the residual system was running on its own, it would approach an extreme position beyond the regular state space due to its special dynamics. In other words, the residual system could not maintain its own regular operating and functionality, thus the deletions of ALDO and R5PI were both regarded as having large qualitative influence in system dynamics.

Moreover, the system had no equilibrium at all after deleting enzymes such as PGK, ENO or GAPDH (Figure 4E). This meant that the original equilibrium was destroyed and the residual system could not re-establish another one. This was because that the residual system upon the removal of anyone of the three enzymes was so ill-suited that its trajectories did not exhibit the normality of well-imposed biological kinetic systems, in which all trajectories tended to stabilize near some regions in the state space. This also indicated that the residual system, if operating on its own, could not effectively maintain its functionality. Hence, the deletions of PGK, ENO and GAPDH were regarded as having even larger qualitative influences in system dynamics compared with the previously mentioned enzymes.

In contrast to the above, enzymes like PGI, PK and PGM again showed a different property. After deleting anyone of them, the residual system still had an equilibrium locating very near to *X_eq_*. Moreover, this equilibrium was also asymptotically stable, with all dimensions converging to it (Figure 4F). Therefore, PGI, PK or PGM knockout did not qualitatively change the system dynamics. Hence, for system-level properties so far as dynamical stability was considered, enzymes like PGK, ALDO, TKb, etc. were more critical than enzymes like PGI, PK, and PGM. Based on all above, we could see that one class of enzymes exemplified by PGK, ENO, TKb, ALDO, TIS, R5PI, GAPDH, and TA have critical properties in terms of both impact on system states and dynamical stability. And the other class of enzymes exemplified by PGI, PK, and PGM had opposite properties. Therefore, the former class was characterized as "critical" and the latter was "uncritical".

### Comparison with experimental characterizations

**Figure 5:**
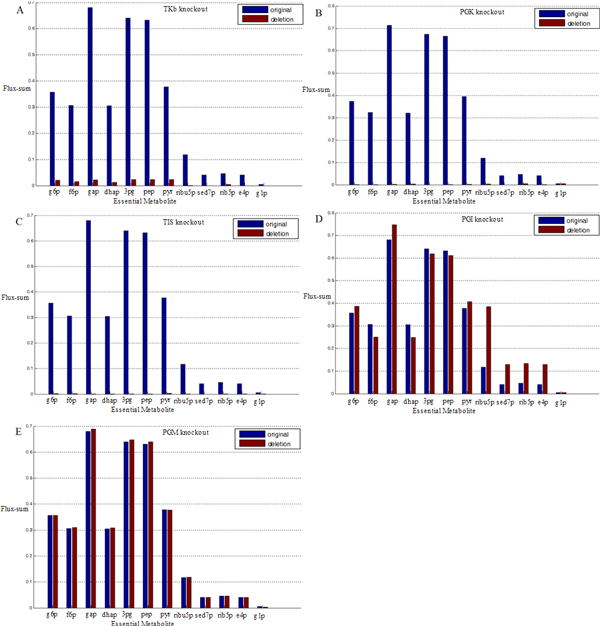
**The flux-sums of essential metabolites before and after enzyme deletions**. The flux-sum values of the 12 essential metabolites before and after enzyme knockout are shown. The x-axis: metabolites; y-axis: the flux-sum values. The blue bars: the flux-sum values in the original system; the red bars: the flux-sum values in the system after enzyme knockout. (A - C) Flux-sums of the essential metabolites upon the knockouts of TKb, PGK and TIS respectively. All metabolites suffer devastating flux reductions. (D - E) Flux-sums of the essential metabolites upon the knockouts of PGI and PGM respectively. All metabolites' flux-sums can be sustained at a high level compared with the original values. The observations support our conclusion that the former enzymes are more critical than the latter ones. See Additional file 6 for abbreviations of metabolites.

We compared our characterizations of system-level properties with characterizations of essentiality from the basis of multiple (previous) validated studies. Kim *et al.*'s work on *E. coli *metabolism defined a set of essential metabolites and demonstrated that if the flux-sum of an essential metabolite reduced by more than 50%, the cell growth rate would decrease by more than 50% correspondingly [6]. There were 12 such metabolites in our working model and we examined their flux-sums by utilizing the simulation power of the kinetic model with respect to perturbations (i.e. enzyme deletion). A naive method was modifying the corresponding enzymatic parameter to zero and leaving the rest of the system as they originally were. However, the theory of Minimization of Metabolic Adjustment (MOMA) suggested that when a severe perturbation occurred, the system adjusted itself to some extent towards a state that was close to normal [16]. Since MOMA was accepted as a rationale, we adopted it in flux simulation upon enzyme deletions, formulating the computation as an optimization problem and solving it numerically (see section "Methods" for details). We found that the flux-sums of the essential metabolites were reduced much more than 50% by deleting any of the enzymes that we predicted as critical (Figure 5A-5C, Additional file 5), thus their deletions would each result in more than 50% reduction in cell growth according to Kim *et al. *On the other side, deleting any of the (predicted) uncritical enzymes did not cause any of the flux-sums to drop by 50% (Figure 5D-5E, Additional file 5), thus they had relatively mild effect on cell growth. This indicated that the predicted critical enzymes had much more weight in functional essentiality than the uncritical enzymes, which well supported our characterizations of criticality.

We also compared our results with other *E. coli *gene essentiality studies such as the Keio collection, the genetic footprinting study and the Profiling of *E. coli *Chromosome (PEC) database, and our results were supported by some of the experimental characterizations. For example, the "critical" enzymes PGK and GAPDH are encoded by genes *pgk *and *gapA *respectively. And the 2 genes are both characterized as essential by studies of both the Keio collection and genetic footprinting [14,19]. ENO is encoded by gene *eno *and this gene is also essential, according to the Keio collection and the PEC database [19,20]. Moreover, the gene fbaA, which encodes ALDO, is characterized as essential by all the Keio collection, genetic footprinting and the PEC database [14,19,20]. Furthermore, the "uncritical" enzymes PGI, PK and PGM are encoded by genes *pgi, pykF *and *pgm *respectively, and the 3 genes are all characterized as nonessential by all the Keio collection, genetic footprinting and the PEC database [14,19,20]. Such comparisons showed that our predictions were consistent with experimental results (Table 1). In addition, ribulose-5-phosphate epimerase (Ru5P) is encoded by gene *rpe*. However, this gene is characterized as essential by genetic footprinting but nonessential by the Keio collection and PEC database. Given that Ru5P is critical to the central carbon metabolic system as revealed by our method and verified by the flux-sums of essential metabolites (earlier context), we propose from the viewpoint of criticality that gene *rpe *might be essential.

**Table 1:** Comparison with multiple *E. coli *gene essentiality studies

Protein	Gene	Criticality	Flux-sum	Keio collection	Genetic footprinting	PEC database
PGK	*pgk*	Critical	√	√	√	
GAPDH	*gapA*	Critical	√	√	√	
ENO	*eno*	Critical	√	√		√
ALDO	*fbaA*	Critical	√	√	√	√
PGI	*pgi*	Uncritical	√	√	√	√
PK	*pykF*	Uncritical	√	√	√	√
PGM	*pgm*	Uncritical	√	√	√	√
Ru5P	*rpe*	Critical	√		√	
G1PAT	*glgC*	Uncritical	√	√	√	√

Our characterizations of protein criticality (Col 3) are compared with multiple gene essentiality studies (Col 5-7) besides the validations through flux-sum (Col 4).

Note: "√" means this evidence supports the characterization of criticality

## Discussion

Studying system-level properties of bio-molecules is essential to systems biology [1,2]. But most studies are based on either network topology that is not working very well at the protein level, or flux analysis that lacks in system level perspective [13,14,21]. To overcome such drawbacks, we propose a method of criticality characterization on the basis of kinetic modeling. In a kinetic system, every interaction is expressed by a kinetic rate equation. How a component influences the system is determined by both its position and the kinetic parameters. Position is equivalent to topological property, while kinetic parameters encode specific biochemical/biological functions. Both kinds of information are integrated in modeling and revealed by dynamical simulation [15,22]. According to the typical formulism of biochemical systems, the kinetic rate equations constitute the deterministic part of the complex system dynamics and they can be viewed as the "driving force" of the system [23]. Thus theoretically, the criticality characterization proposed in our method is the study of structural factors built into the "driving force" of a system.

Differing from topology-based methods, our method characterizes system-level properties on the quantitative basis. But unlike the conventional sensitivity analysis, we employ the network structure information by calculating the distances from the deleted spot to the affected entities besides computing the fluctuations. Moreover, unlike conventional flux-analysis approaches, we explore the system stability and retrieve system dynamics structure. Incorporating the network/dynamics structure information allows us to reveal the simultaneous/collateral influences and the overall impact on the system. Another major difference from the sensitivity analysis is that we use *in silico *deletions instead of mild perturbations (e.g. 5% or 10%, as most flux-analysis approaches do). Because a well-casted biological network usually has parametric properties favouring the robustness in dynamics, critical components may well tolerate mild perturbations (i.e. parameters exhibiting the Lyapunov stability). Therefore, individual sensitivity analysis often fails to identify such critical spots, and its inability to reveal simultaneous influences worsens the situation. That is why we develop the "criticality characterization". *In silico *deletion is equivalent to investigating how the system would be if the component is forcefully assumed to be absent, eliminating the parametric properties stated earlier. Furthermore, our method's capacity of revealing simultaneous/overall impacts at the system level enables it to distinguish real critical spots from uncritical ones more effectively. In addition, utilizing kinetic model as the analytical basis is a superiority over the stoichiometric flux-balance modeling in traditional flux-analysis methods, enabling us to appropriately explore system behaviours in the real-time scale [15]. For example, both traditional topology-based and flux-analysis approaches regard TIS as peripheral as it is not highly connected and it is not on any uni-directional or rate limiting reactions. However, there were experimental studies showing that knocking out *tpiA *(i.e. the gene encoding TIS) attenuated the cell growth by about 70%. And our method appropriately revealed that TIS could exert large impacts on the system if deleted, because of the designs we made (mentioned above). Hence methodologically, our method creates a different angle from topology-based methods and can be viewed as an improvement of conventional flux-analysis approaches.

After *in silico *deleting a protein, the residual system is actually a virtual structure. We assume that this structure encodes important information about whether the mutant can maintain its functionality and how it would dynamically behave/evolve provided that it stills operates on its own. The residual system fails to maintain functionality when its kinetics goes beyond regular ranges (e.g. occurring negative values, or soaring to extreme values exceeding regular intracellular molecular concentrations), or its dynamics is trapped in a mode where the stable equilibrium is sabotaged, as stable equilibrium gives rise to robustness and is an essential prerequisite for valid mathematical formulations of living cell dynamics [17,24-26]. Either case indicates that deleting the protein makes the system so ill-suited that it cannot run on its own.

By applying our method to *E. coli *central carbon metabolism, we find that deleting enzymes such as PGK, GAPDH, etc. causes the system to become a very ill-suited structure as some state values soaring to levels beyond the normal range and the trajectories are highly divergent throughout the state space (Figure 2B and 4B). Likewise, deleting enzymes such as TKb, ALDO, etc. also causes relatively large impacts on both system kinetics and qualitative dynamics (Figure 2A and 4A). On the contrary, knocking out enzymes such as PGI, PK, etc. exerts very small influences (Figure 2D and 4E). We also find enzymes can mediate large influences on distant metabolites or enzymes. For instance, TKb, PGK, PGI, etc. can all exert the largest impacts on entities of distances other than 1 (Figure 2A, 6A-6B and Additional file 2, 3). This is because bio-systems have complex structures consisting of branches, alternative pathways and loops, as well as various kinetic parameters differing in orders of magnitudes [6,24]. Such structure acts as a special leverage, determining special ways of interactions and influence propagations. Only kinetic modeling can reveal such knowledge, and such analyses can give us more clues on selecting potential regulatory targets for use in drug development, metabolic engineering, etc.

**Figure 6:**
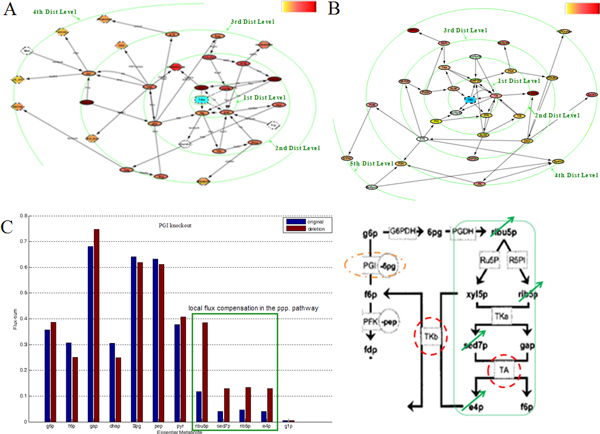
**Remote impact and local flux compensation**. A system component can affect distant entities even greater than its closest neighbours, which is illustrated by TKb. Moreover, asymmetry in the criticalities of components can result in local flux compensation, which is illustrated by the alternative paths of TKb, TA and PGI. (A) An explicit demonstration of Figure 2A in the biological network overlay. It is organized as a metabolite network with TKb highlighted in blue and metabolites arranged along green circles representing distance levels. The amplitude is proportional to the color gradient (upper-right corner, "red-yellow" corresponds to "strong-slight"). (B) The enzyme-centric view of (A). Subfigure (A) and (B) mainly show the distributions of distances and amplitudes in a network's view, whereas the exact vertices' labels are not important here. To see the two pictures in high resolution, refer to Additional file 7 and 8. (C) Metabolites *ribu5p, sed7p, rib5p *and *e4p *in the pentose-phosphate pathway have increased fluxes after PGI knockout due to the asymmetry of system-levels properties of PGI, TKb and TA. The three enzymes mutually form alternative paths associating to the essential metabolite *f6p*.

By utilizing the power of kinetic model for approaching real-time events, we simulated fluxes after enzyme deletions and compared the results with a previously validated study of metabolic essentiality [6]. The comparison shows that our characterization of criticality is well supported by functional essentiality. Interestingly, we discovered that the asymmetry in criticalities of building blocks might give rise to local flux compensation. For instance, multiple metabolites (e.g. ribulose-5-phosphate, sedoheptulose-7- phosphate, etc.) in the pentose phosphate pathway have increased flux-sums after PGI knockout (Figure 5D and 6C). The cutoff of PGI induces the two alternative pathways for generating the essential metabolite fructose-6-phosphate, TKb and TA, to operate at a greater volume. Thus fluxes through relative reactions are compensated, resulting in local amplified fluxes. This is a likely result in accordance to the MOMA mechanism [17]. Although MOMA can compensate system fluxes/states to some degrees, our results show that the effects caused by deletions of critical components such as TKb, TA, PGK, etc. cannot be smoothed by such compensations (Figure 5A-5B, Additional file 5). This is because such compensations are mainly mediated by alternative pathways [6]. When a critical component is deleted, leaving inferior components as backup to rely on, the system cannot work efficiently. On the contrary, deleting PGI leaves its two alternative pathways that are of superior properties at the "ON" state and the system still works, thus fluxes/states can be efficiently compensated. This gives a hint on how criticality characterization can help in bio-system modifications such as in metabolic engineering. We can delete some system components with inferior properties, leaving alternative pathways with superior properties to work. And phenotypes in the local areas relating to such alternative pathways might be compensated due to the leverage of system structure and the MOMA mechanism. Therefore, comprehensive methods of exploring system-level properties can help us make use of bio-complexity in engineering, as well as in knowledge discovery.

It is noteworthy that functionally important components are not necessarily critical, as studies suggest that the more important a reaction is in function, the more likely that it has a backup pathway [6,13]. For example, PK connects very fundamental chemical compounds but it is regarded as uncritical at the system level, because there are alternative paths (e.g. the phosphotransferase system - PTS, in bacteria glycolysis; Additional file 1) that can prevent large impacts on system kinetics/dynamics. This exemplifies that bio-system components have dichotomy. They have "importance" as biochemical molecules, and they also have "criticality" to the system as constitutive building blocks. Actually, our method does not aim to find the "functionally important" molecules, but those "critical" to the system, i.e. components that cannot be absent, or the system will be severely aberrant. Since the criticality of an enzyme depends on many factors (e.g. kinetic parameters, substrates inhibiting/activating other reactions, degree of the effects, etc.), the assignments of system boundaries in modeling might affect prediction results. As the enzymes located on the boundary might have incomplete interplay structure, the above factors may not occur properly in the kinetic equations. Therefore, accurate criticality characterization is facilitated by appropriate system inclusiveness in modeling. For example, glucose-1-phosphate adenyltransferase (G1PAT) only connects the external polysaccharide synthesis pathway, with few interactions with large-capacity reactions both in the system and outside pathways. Thus as the boundary is assigned up to it, the validity of the results are enhanced (Table 1). Furthermore, fundamental, common and conserved pathways must be chosen for comparison with genome-scale gene essentiality studies that regard to global cellular functionality. For instance, the bacteria central carbon metabolism here is an appropriate example [5,7,8], thus various predictions of protein criticality are well consistent with global gene essentiality characterizations [13,14,19].

Although we used a metabolic system as the working model, the application of our method is not confined to metabolic systems. For instance, we can model gene transcription dynamics by deriving gene transcription rate with the power-law formulism, the Hill equation, or equations of chemical kinetic actions [27-29]. Or we can describe ligand-receptor and protein-protein binding actions with the mass action law and build models for signaling networks [22]. We even do not have to obtain exact parameters fitting the modeled solutions to assay measurements when analyzing the generic behavioral potential of the system, e.g. in what parameter ranges the system exhibits certain dynamics and how they change with parameters. Such qualitative predictions are also useful in revealing general principles governing complex bio-systems. Naturally, complicated bifurcation dynamics will be harder to analyze; but the idea of our method can be well applied once the coexisting dynamical characteristics in bifurcation are associated with biological implications [28]. By integrating knowledge and using theoretical generic forms of models [15,30], kinetic modeling will be eventually feasible for more organisms. Hence, instead of the traditional approaches, we propose that complex systems be studied by casting the network into kinetic equations and computing the system-level properties with respect to system kinetics/dynamics (criticality). Overall, our method may provide a new viewpoint in revealing constitutive/functional properties of building blocks in a biological system.

## Conclusions

Our method creates a new angle from traditional topology-based methodologies for evaluating system-level properties of bio-molecules. Moreover, the proposed method can be viewed as an improvement of the conventional flux-analysis approaches such as FBA and MCA. In addition, the method leads to results that are consistent with experimental references, showing that it is efficient in characterizing protein criticality and studying biological systems. Furthermore, the method's application can be extended to other types of bio-systems (e.g. transcriptional networks and signaling networks) to reveal the constitutive/functional properties of system building blocks.

## Methods

### Kinetic modeling

We utilized existing kinetic data in *E. coli *central carbon metabolism and adopted a previous modeling framework as our working platform [5]. The kinetic model consists of 30 metabolites (including external metabolites and biosynthesis products) and 30 biochemical reactions (24 enzymes and 6 lumped reactions standing for transport/biosynthetic processes relating to external pathways; Additional file 1). The model can also be re-casted into an enzyme-centric network, by adding a directed connection from enzyme A to B if any of A's products was B's substrate. We could explicitly see the interactions among enzymes from the enzyme-centric view (Additional file 1).

All kinetic rate equations were formulated according to biochemical mechanisms [5]. Most of them were casted in the uni-/bi-substrate Michaelis-Menten formulism. The kinetics for each metabolite was expressed by an ordinary differential equation (ODE; Eqn (1)).

(1) $$\frac{dX}{dt}=A\cdot R\left(X,P\right)+B\left(X,P\right)$$

Here vector *X *denoted system state and *P *denoted kinetic parameters. *R *was a function vector collocated by all rate equations, and *A *was the stoichiometric matrix. *B *was the term standing for extra reactions (e.g. transport, metabolite utilization for cellular growth, etc). Most parameters were found in published studies and the rest could be estimated using the experimental conditions, steady-state reaction rates and concentrations reported in previous studies [5,31,32]. For complete descriptions of metabolites, reactions, forms of kinetic rate equations and ODEs, see Additional file 6.

### Dynamical simulation and state fluctuation

By substituting in an initial value, a typical Cauchy problem was formed and numerical integration curves were computed for Eqn (1). We used the Gear method in computation so as to alleviate the stiffness problem of ODEs [33]. With an initial value for normal experimental conditions [5], we obtained the kinetic states of the system *X^0^*, i.e. time-courses of metabolite concentrations under normal conditions. After deleting an enzyme, we computed the kinetics of the residual system *X^e ^*to see how it deviated from the original state. Thus the influence of the deleted enzyme could be assessed. Assuming solution *X *was organized as a matrix and each column represented the kinetics of a metabolite, we could calculate the amplitude of metabolite *k*'s state fluctuation as

(2) $$f_{k}=||X_{∙k}^{e}-X_{∙k}^{0}|{|}_{2}/||X_{∙k}^{0}|{|}_{2}$$

We could calculate the distances of metabolites from the deleted enzyme by the structure of metabolite-centric network. Metabolites directly associating with the enzyme were assigned a distance of 1; metabolites not directly associating with the enzyme but associating with the 1^st ^distance level metabolites within a direct single reaction were assigned a distance of 2, and so on. We combined the distances and amplitudes to see in which ranges influences occurred and how strong they were. We also computed the flux distributions of the residual system based on the metabolites concentrations and rate equations. Thus we could observe how the flux distributions deviated from the original system and assessed them in the same way as Eqn (2). The distances of effects could be directly counted from the enzyme-centric network. Furthermore, we could combine the amplitude and distance data into a single measurement for assessing the overall impact, both for metabolite-centric network and enzyme-centric network (Eqn (3)).

(3) $$M\left(d,f\right)=\sqrt{\underset{k}{\sum }f_{k}d_{k}^{n}},n\in N_{+}$$

### Dynamical stability

Normal bio-systems are subjected to robustness as they structurally consist of abundant alternative pathways and feedback loops [6,17,24]. Thus valid formulations of bio-systems usually have stable equilibrium, attracting neighborhood trajectories and allowing slight changes to be tolerated without disturbing normality [5,25,26]. The trajectories tend to some area over adequately large ranges of time and parameter spaces if the system has equilibrium. And if it did not, trajectories spread out along some dimensions traversing several orders of magnitudes. To locate the equilibrium, we utilized the state at the end time point of simulation as an initial guess and used the trust-region method to solve the problem [26,34]. By carefully refining the numerical approach, the equilibrium could be computed and distances from the original *X_eq _*were calculated by the Euclid norm.

We defined the dynamical stability following the concept of the Lyapunov stability, which has explicit physical/chemical context and is suitable for describing metabolic robustness [25,26]. The stability of equilibrium is determined by the eigenvalues of the Jacobian matrix evaluated at the equilibrium (Eqn (4)). If all eigenvalues have negative real parts, the equilibrium is asymptotically stable; if any of them has a positive real part, the equilibrium is unstable; and if the Jacobian matrix has a pair of purely imaginary conjugate eigenvalues, a limit cycle is likely to bifurcate out of the equilibrium.

(4) $$J_{X_{eq}}={\left[\frac{\partial \left(A\cdot R\left(X,P\right)\right)}{\partial X}\right]}_{X=X_{eq}}$$

The Hartman-Grobman Theorem and Center Manifold Theorem prove that if the Jacobian matrix evaluated at an equilibrium has 2 conjugate purely imaginary eigenvalues, *N_s _*eigenvalues with negative real parts and *N_u _*eigenvalues with positive real parts, the trajectories of Eqn (1) near the equilibrium are topologically equivalent to those of Eqn (5). Here β is a part of kinetic parameters and σ is +1 according to our system. In other words, the orbit structure (near the equilibrium) of Eqn (5) is topologically conjugate with that of Eqn (1). Because Eqn (5) is much simpler, we could investigate it instead of the complex Eqn (1). In this way, we explicitly drew the orbit structure of Eqn (5) near the equilibrium and could know the qualitative system dynamics of Eqn (1) accordingly.

(5) $$\left\{\begin{array}{c} \frac{dy_{1}}{dt}=\beta y_{1}-y_{2}+\sigma y_{1}\left(y_{1}^{2}+y_{2}^{2}\right)\\ \frac{dy_{2}}{dt}=y_{1}+\beta y_{2}+\sigma y_{2}\left(y_{1}^{2}+y_{2}^{2}\right)\\ \frac{dy^{N_{s}}}{dt}=-y^{N_{s}}\\ \frac{dy^{N_{u}}}{dt}=y^{N_{u}} \end{array}\right)$$

If the bifurcation caused by an *in silico *deletion (parameter modification) yields multiple equilibriums, the impact on dynamical stability is regarded as large if anyone of the equilibriums exhibit qualitative difference from *X_eq _*in dynamical properties.

### MOMA and flux-sum

MOMA suggested that metabolic systems were subjected to biological robustness. When perturbed, it could adjust itself towards a state that was relatively close to the original state. We could formulate the process as an optimization problem as

(6) $$\begin{array}{c} \text{min}\;S\left(P_{\mu }\right)={∥X\left(P_{\mu }\right)-X_{0}∥}_{2}\\ \text{s}\text{.t}\text{. }\left\{\begin{array}{c} \frac{dX}{dt}=A\cdot R\left(X,P_{\mu }\right)\\ \overset{⇀}{0}\leq X\\ \overset{⇀}{0}\leq P_{\mu }\\ X\left(t_{0}=0\right)=C_{0} \end{array}\right) \end{array}$$

Here *P_μ _*was the parameter set with the relevant enzymatic parameters deleted, *X_0 _*was the original state and *C_0 _*was the initial value. Some states that were close to *X_0 _*in the feasible space could be solved with the genetic algorithm, a heuristic numerical approach that can alleviate computation difficulty in large variable space to some extent.

We adopted the definition of essential metabolite and flux-sum in Kim *et al.*'s work on *E. coli *metabolism [6]. The 12 essential metabolites occurred in central carbon metabolism were shown in Figure 5. Here the flux-sum of metabolite *k *was defined as

(7) $$\Phi_{k}=\underset{i\in \Omega_{k}}{\sum }A_{ki}\cdot R_{i}\left(X,P\right)$$

where *Ω_k _*was the index set of reactions producing metabolite *k*.

After MOMA computation, we obtained one (or more) set of parameters and system states. Using rate equations, we simulated the fluxes and calculate flux-sums according to Eqn (7).

## Competing interests

The authors declare that they have no competing interests.

## Authors' contributions

Conceiving and designing the research: RDL and LL. Data acquisition and analysis: RDL. Drafting the manuscript: RDL and LL.

## Supplementary Material

Additional file 1 **The metabolite-centric and enzyme-centric views of the *E. coli *central carbon metabolic network**. The file is in the format of *.png, with figures included showing the metabolite-centric (Page 1) and enzyme-centric metabolic (Page 2) networks. In the metabolite-centric view, enzymes/reactions are abbreviated as symbols and denoted by rectangles; metabolites are also abbreviations; inhibitors/activators are drawn as circles beside the reactions. Synth1 is a lumped reaction for synthesizing chorismate and murine; Synth2 is lumped reaction for synthesizing isoleucine, alanine, ketoisovalerate, and diaminopimelate [5]. In the enzyme-centric view, enzymes are denoted by circles and arrows indicate interactions. For more detailed information, see the "Methods" section and Additional file 6. Click here for file

Additional file 2 **Impacts on system states in the metabolite-centric view**. The file is in the format of *.pdf, with each plot showing the impacts of enzymes deletions on the metabolite kinetics. The metabolite indexes, impact distances and state uation amplitudes form the 3 dimensions. Legends are the same as those in Figure 2. Click here for file

Additional file 3 **Impacts on system states in the enzyme-centric view**. The file is in the format of *.pdf, with each plot showing the impacts of enzymes deletions on the kinetic fluxes. The reaction/enzyme indexes, impact distances and flux fluctuation amplitudes form the 3 dimensions. Legends are the same as those in Additional file 2. Click here for file

Additional file 4 **The catalogue of the results of dynamical stability analysis**. The file categorizes all enzymes that generate topologically equivalent system orbit structures, when deleted. Enzymes in the same category exert similar impacts on the qualitative dynamics of the system. The file is in the format of MS Word electronic table (*.doc). Click here for file

Additional file 5 **The flux-sum validations of critical and uncritical enzymes**. The file is in the format of *.pdf, with each plot showing the flux-sums of the essential metabolites before and after the deletion of an enzyme. Deletions of all presented enzymes are shown. The metabolite symbols and flux-sum values form the lateral and vertical dimensions. Legends are the same as those in Figure 5. Click here for file

Additional file 6 **Description of the modeling**. The file is the supplementary text of the modeling. It is in the format of *.doc and includes detailed descriptions of metabolites, enzymes, kinetic rate equations and ODEs. Click here for file

Additional file 7 **High resolution images of **Figure 6A and 6B. The two files (in the format of *.png) are the high resolution versions of Figure 6A and 6B, respectively. Click here for file

Additional file 8 **High resolution images of **Figure 6A and 6B. The two files (in the format of *.png) are the high resolution versions of Figure 6A and 6B, respectively. Click here for file

## References

[B1] Hood L Systems biology: Integrating technology, biology and computation Mech Ageing Dev 2003 124 9 16 10.1016/S0047-6374(02)00164-1 12618001

[B2] Ideker T Galitski T Hood L A new approach to decoding life: Systems biology Annu Rev Genomics Hum Genet 2001 2 343 372 10.1146/annurev.genom.2.1.343 11701654

[B3] Bailey JE Toward a science of metabolic engineering Science 1991 252 1668 1675 10.1126/science.2047876 2047876

[B4] Li H Zhan M Systematic intervention of transcription for identifying network response to disease and cellular phenotypes Bioinformatics 2006 22 96 102 10.1093/bioinformatics/bti752 16278241

[B5] Chassagnole C Noisommit-Rizzi N Schmid JW Mauch K Reuss M Dynamic modeling of the central carbon metabolism of *Escherichia coli* Biotechnol Bioeng 2002 79 53 73 10.1002/bit.10288 17590932

[B6] Kim PJ Lee DY Kim TY Lee KH Jeong H Lee SY Park S Metabolite essentiality elucidates robustness of *Escherichia coli *metabolism Proc Natl Acad Sci USA 2007 104 13638 13642 10.1073/pnas.0703262104 17698812 PMC1947999

[B7] Neidhardt FC Curtiss R Ingraham JL Lin ECC Low KB Magasanik B Reznikoff W Riley M Umbarger HE Escherichia coli and Salmonella: Cellular and molecular biology 1996 Washington, DC: ASM Press

[B8] Milo R Itzkovitz S Kashtan N Levitt R Shen-Orr S Ayzenshtat I Sheffer M Alon U Superfamilies of Evolved and Designed Networks Science 2004 303 1538 1542 10.1126/science.1089167 15001784

[B9] Ma H Zeng AP Reconstruction of metabolic networks from genome data and analysis of their global structure for various organisms Bioinformatics 2003 19 270 277 10.1093/bioinformatics/19.2.270 12538249

[B10] Covert MW Palsson BO Transcriptional Regulation in Constraints-based Metabolic Models of *Escherichia coli* J Biol Chem 2002 277 28058 28064 10.1074/jbc.M201691200 12006566

[B11] Jeong H Tombor B Albert R Oltvai ZN Barabasi AL The large-scale organization of metabolic networks Nature 2000 407 651 654 10.1038/35036627 11034217

[B12] Vitkup D Kharchenko P Wagner A Influence of metabolic network structure and function on enzyme evolution Genome Biol 2006 7 R39 10.1186/gb-2006-7-5-r39 16684370 PMC1779518

[B13] Ghim CM Goh KI Kahng B Lethality and synthetic lethality in the genome-wide metabolic network of *Escherichia coli* J Theor Biol 2005 237 401 411 10.1016/j.jtbi.2005.04.025 15975601

[B14] Gerdes SY Scholle MD Campbell JW Balazsi G Ravasz E Daugherty MD Anderson I Gelfand MS Bhattacharya A Kapatral V D'Souza M Baev MV Grechkin Y Mseeh F Fonstein MY Overbeek R Barabasi AL Oltvai ZN Osterman AL Experimental Determination and System Level Analysis of Essential Genes in *Escherichia coli *MG1655 J Bacteriol 2003 185 5673 5684 10.1128/JB.185.19.5673-5684.2003 13129938 PMC193955

[B15] Li RD Li YY Lu LY Ren C Li YX Liu L An improved kinetic model for the acetone-butanol-ethanol pathway of *Clostridium acetobutylicum *and model-based perturbation analysis BMC Syst Biol 2011 5 S12 21689471 10.1186/1752-0509-5-S1-S12 PMC3121112

[B16] Segrè D Vitkup D Church GM Analysis of optimality in natural and perturbed metabolic networks Proc Natl Acad Sci USA 2002 99 15112 15117 10.1073/pnas.232349399 12415116 PMC137552

[B17] Kitano H Biological robustness Nat Rev Genet 2004 5 826 837 15520792 10.1038/nrg1471

[B18] Wagner A Robustness and evolvability in living systems 2005 Princeton: Princeton University Press

[B19] Baba T Ara T Hasegawa M Takai Y Okumura Y Baba M Datsenko KA Tomita M Wanner BL Mori H Construction of *Escherichia coli *K-12 in-frame, single-gene knockout mutants: the Keio collection Mol Syst Biol 2006 2 2006.0008 10.1038/msb4100050 PMC1681482 16738554

[B20] Hashimoto M Ichimura T Mizoguchi H Tanaka K Fujimitsu K Keyamura K Ote T Yamakawa T Yamazaki Y Mori H Katayama T Kato J Cell size and nucleoid organization of engineered *Escherichia coli *cells with a reduced genome Mol Microbiol 2005 55 137 149 15612923 10.1111/j.1365-2958.2004.04386.x

[B21] Desai RP Harris LM Welker NE Papoutsakis ET Metabolic flux analysis elucidates the importance of the acid-formation pathways in regulating solvent production by *Clostridium acetobutylicum* Metab Eng 1999 1 206 213 10.1006/mben.1999.0118 10937935

[B22] Lee JM Gianchandani EP Eddy JA Papin JA Dyanmic analysis of integrated signaling, metabolic, and regulatory networks PLoS Comput Biol 2008 4 e1000086 10.1371/journal.pcbi.1000086 18483615 PMC2377155

[B23] Ao P Metabolic network modeling: Including stochastic effects Comput Chem Eng 2005 29 2297 2303 10.1016/j.compchemeng.2005.05.007

[B24] Kim D Kwon YK Cho KH Coupled positive and negative feedback circuits form an essential building block of cellular signaling pathways BioEssays 2007 29 85 90 10.1002/bies.20511 17187378

[B25] Kuznetsov YA Elements of applied bifurcation theory, Springer 2004 3

[B26] Strogatz SH Nonlinear dynamics and chaos: with applications to physics, biology, chemistry, and engineering 2001 Westview Press

[B27] Savageau MA Voit EO Recasting nonlinear differential equations as S-systems: a canonical nonlinear form Math Biosci 1987 87 83 115 10.1016/0025-5564(87)90035-6

[B28] Huang S Guo YP May G Enver T Bifurcation dynamics in lineage-commitment in bipotent progenitor cells Dev Biol 2007 305 695 713 10.1016/j.ydbio.2007.02.036 17412320

[B29] Bintu L Buchler NE Garcia HG Gerland U Hwa T Kondev J Phillips R Transcriptional regulation by the numbers: models Curr Opin Genet Dev 2005 15 116 124 10.1016/j.gde.2005.02.007 15797194 PMC3482385

[B30] Lee LW Yin L Zhu X Ao P Generic enzymatic rate equation under living conditions J Biol Syst 2007 15 495 514 10.1142/S0218339007002295

[B31] Bhattacharya M Fuhrman L Ingram A Nickerson KW Conway T Single-run separation and detection of multiple metabolic intermediates by anion-exchange high-performance liquid chromatography and application to cell pool extracts prepared from *Escherichia coli* Anal Biochem 1995 232 98 106 10.1006/abio.1995.9954 8600840

[B32] Buziol S Bashir I Baumeister A Claaßen W Noisommit-Rizzi N Mailinger W Reuss M New bioreactor-coupled rapid stopped-flow sampling technique for measurements of metabolite dynamics on a subsecond time scale Biotechnol Bioeng 2002 80 632 636 10.1002/bit.10427 12378604

[B33] Hairer E Wanner G Solving ordinary differential equations II: Stiff and differential-algebraic problems 1996 2 Berlin: Springer-Verlag

[B34] Conn AR Gould NIM Toint PL Trust-region methods MPS/SIAM Series on Optimization. Society for Industrial Mathematics 1987

