# Supplementary figures and images for: Characterizing criticality of proteins by systems dynamics: Escherichia coli central carbon metabolism as a working example
Source: BMC Syst Biol. 2012 Jul 16;6(Suppl 1):S11. doi: 10.1186/1752-0509-6-S1-S11 (PMC3402961; doi:10.1186/1752-0509-6-S1-S11)

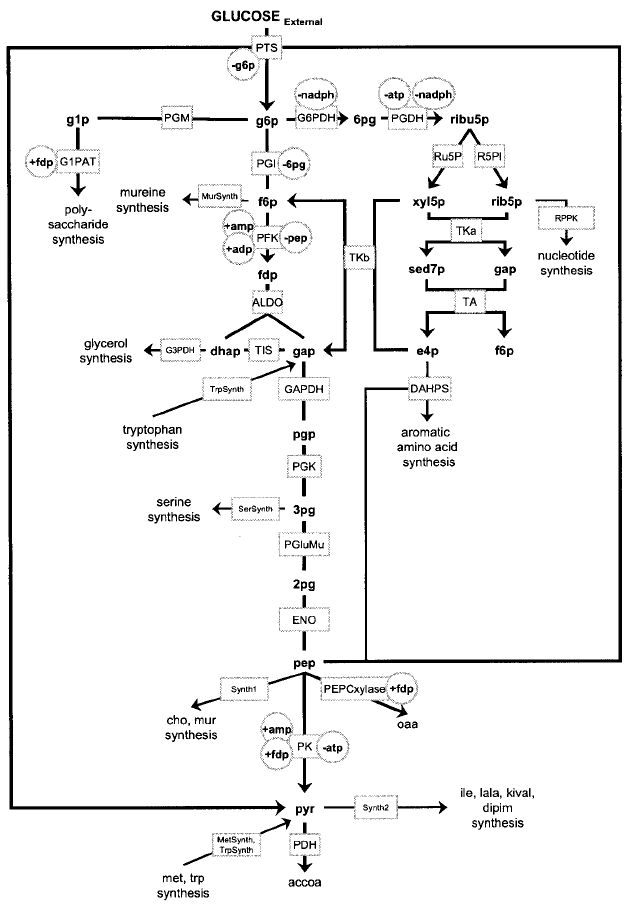

Supplement: Additional file 1 — The metabolite-centric and enzyme-centric views of the E. coli central carbon metabolic network. The file is in the format of *.png, with figures included showing the metabolite-centric (Page 1) and enzyme-centric metabolic (Page 2) networks. In the metabolite-centric view, enzymes/reactions are abbreviated as symbols and denoted by rectangles; metabolites are also abbreviations; inhibitors/activators are drawn as circles beside the reactions. Synth1 is a lumped reaction for synthesizing chorismate and murine; Synth2 is lumped reaction for synthesizing isoleucine, alanine, ketoisovalerate, and diaminopimelate [5]. In the enzyme-centric view, enzymes are denoted by circles and arrows indicate interactions. For more detailed information, see the "Methods" section and Additional file 6. [file 1752-0509-6-S1-S11-S1.png]

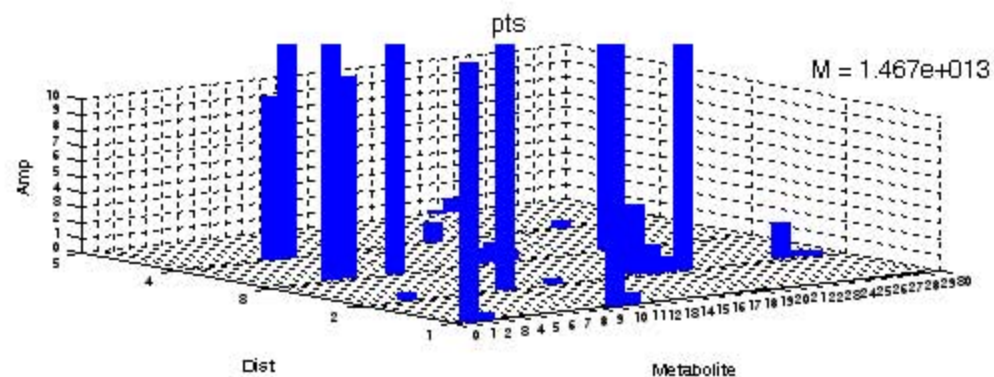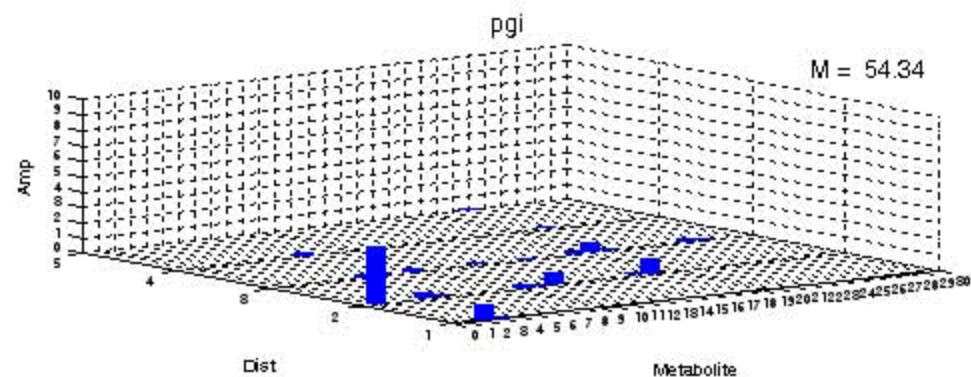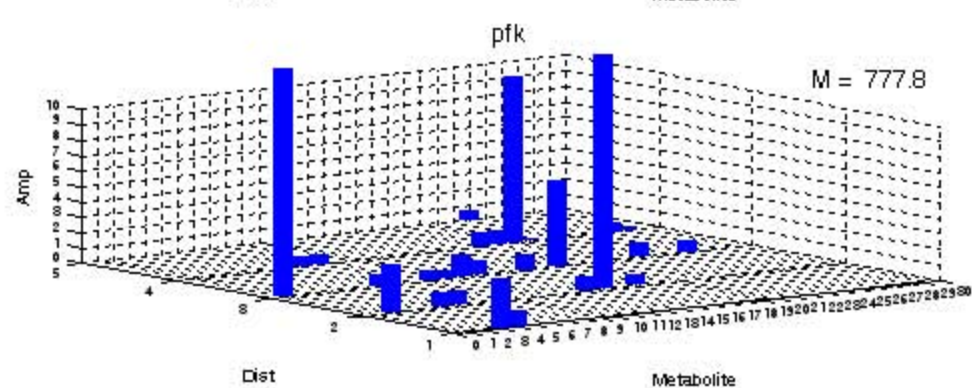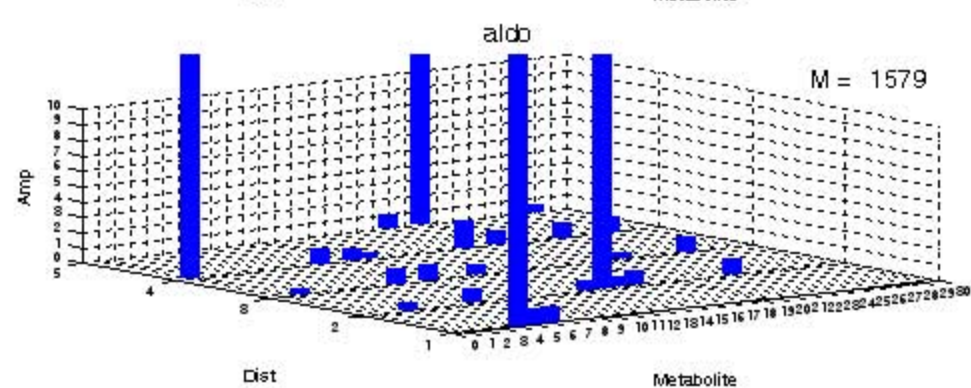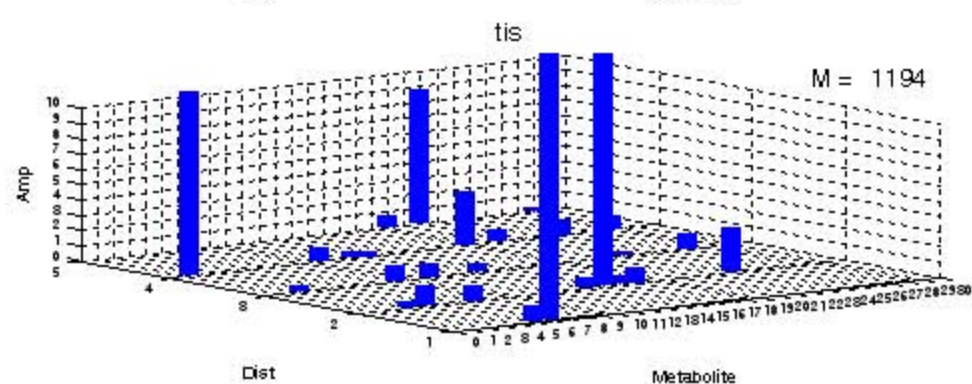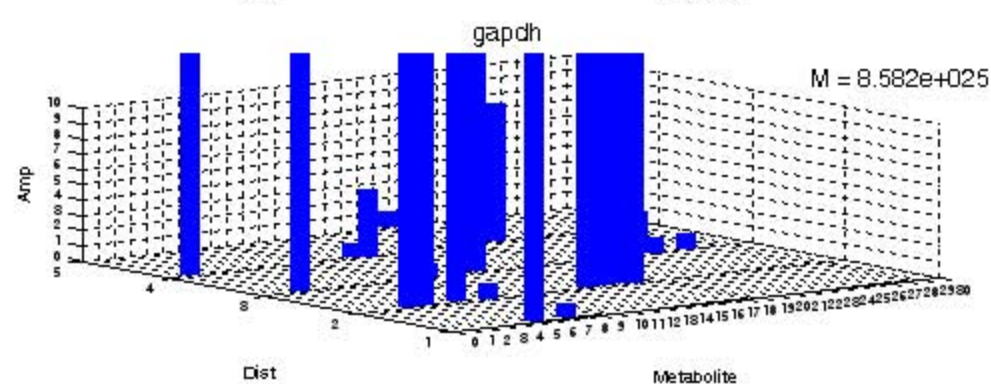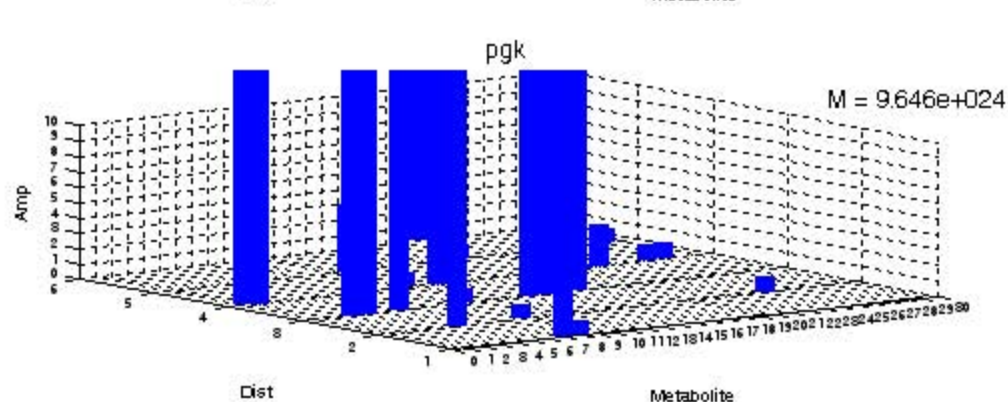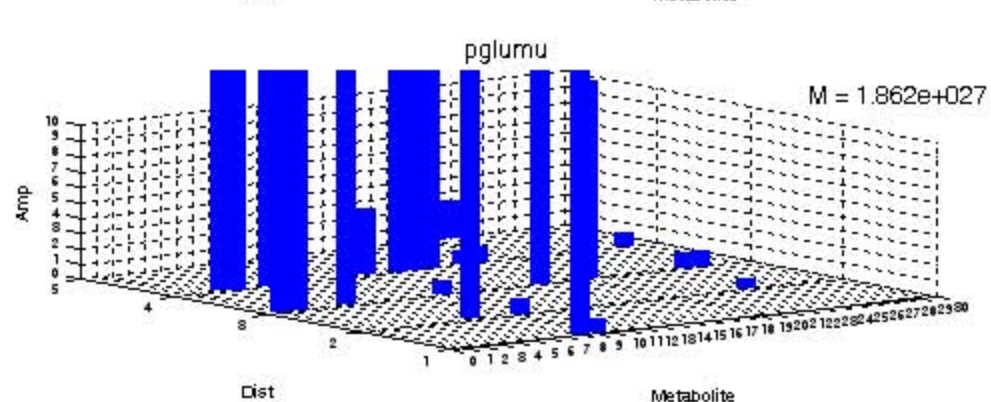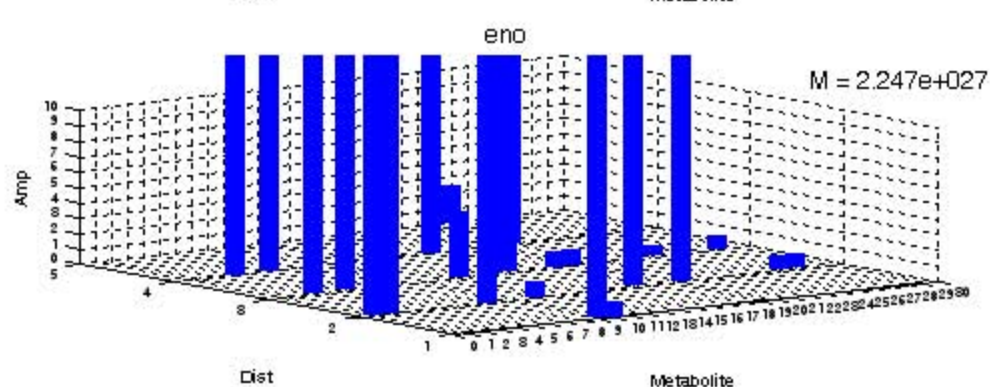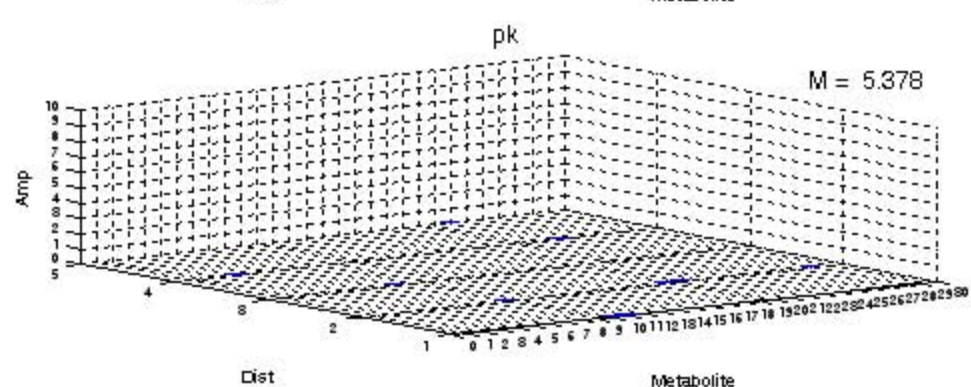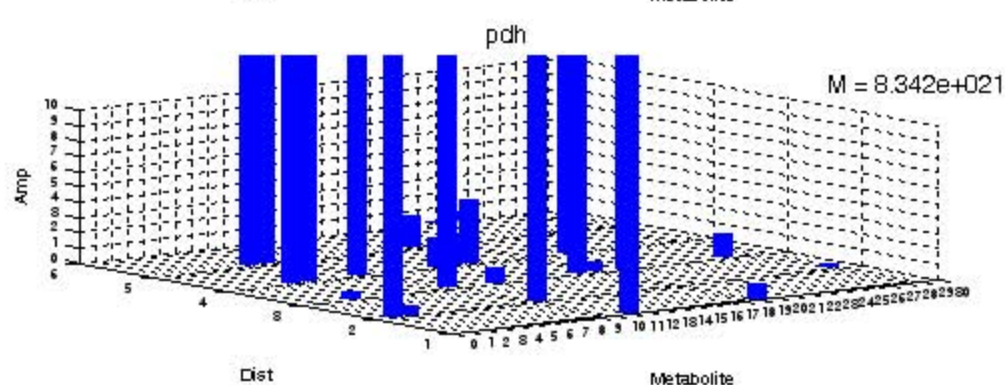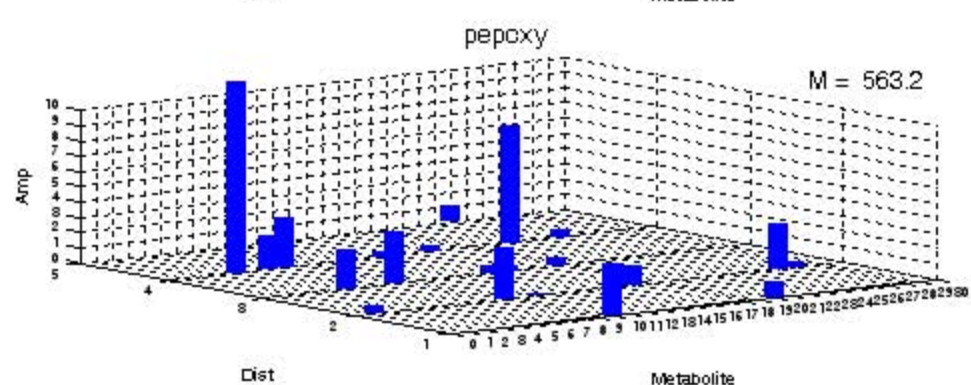

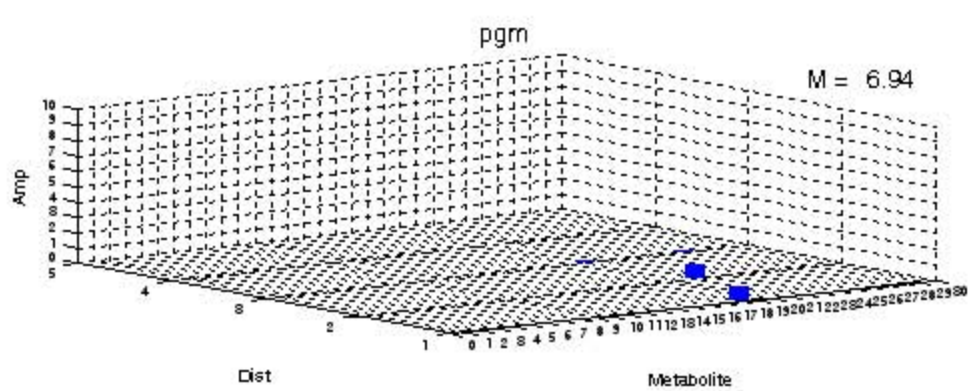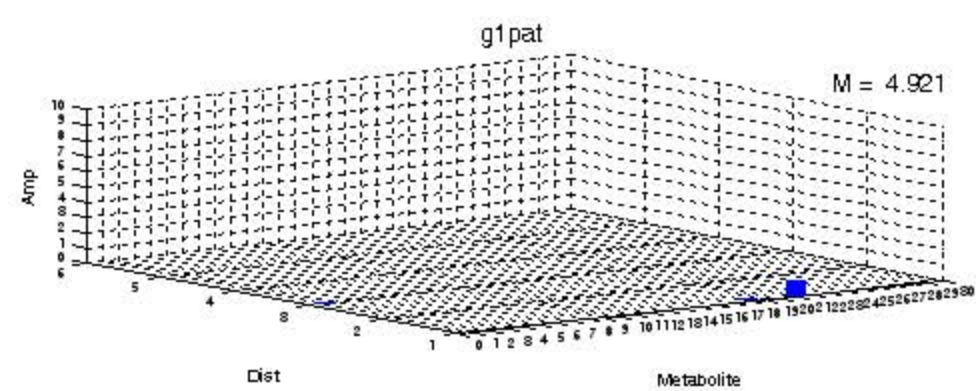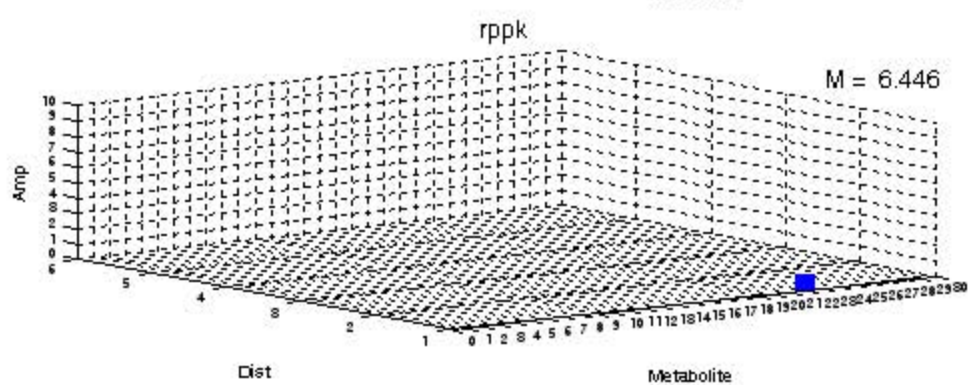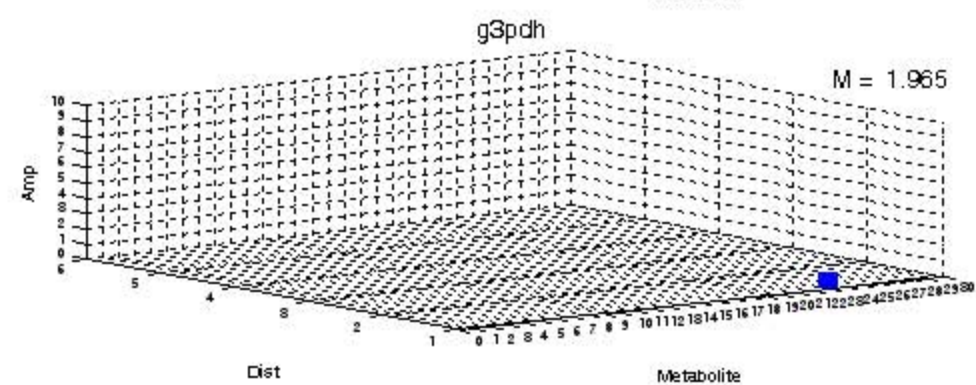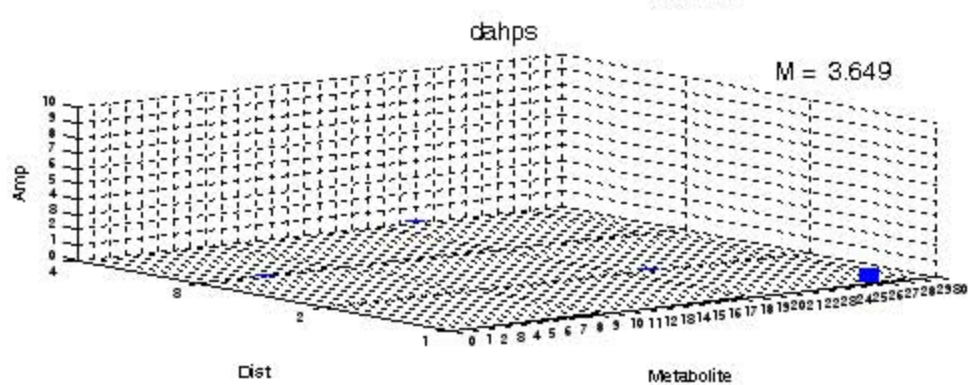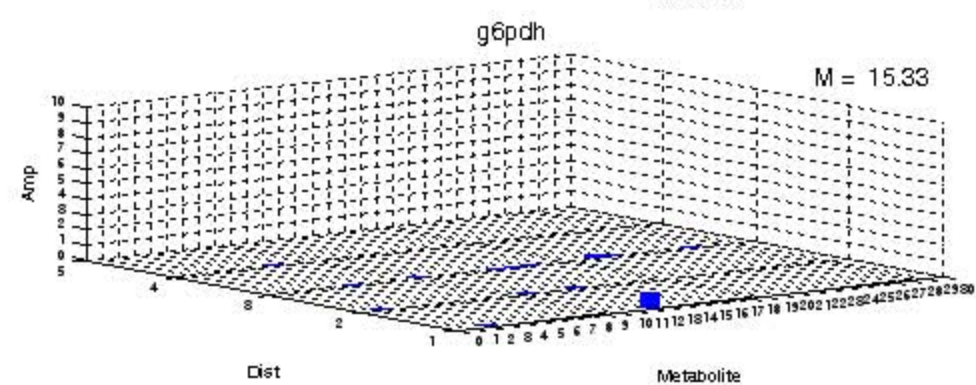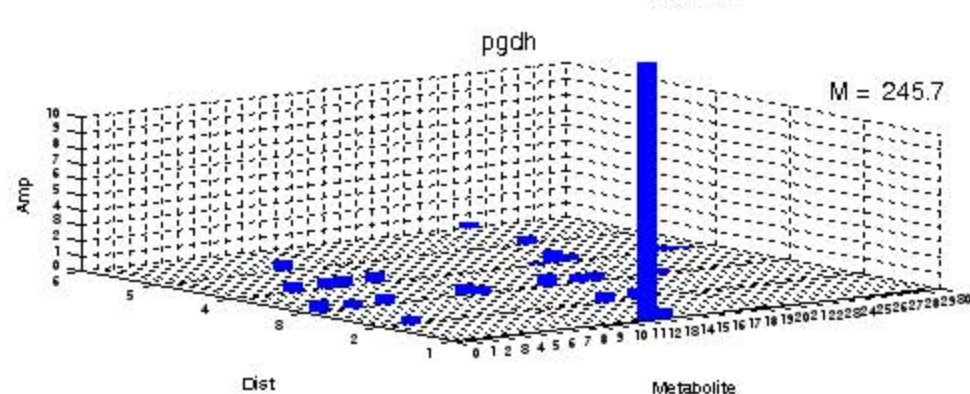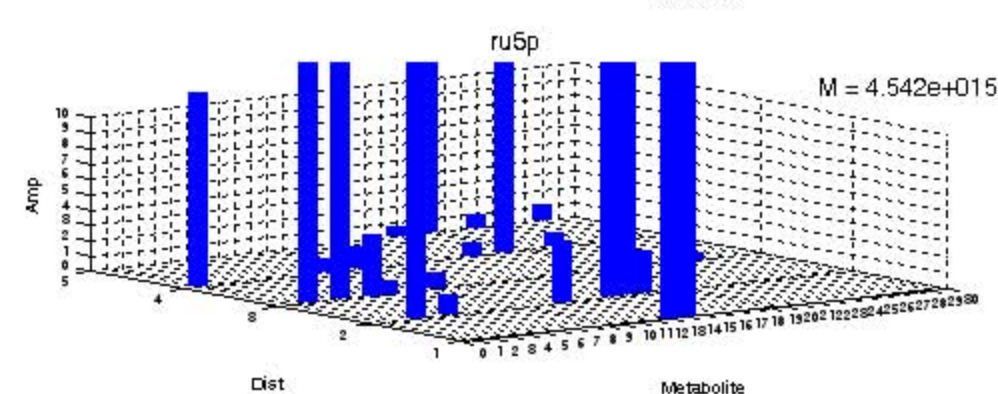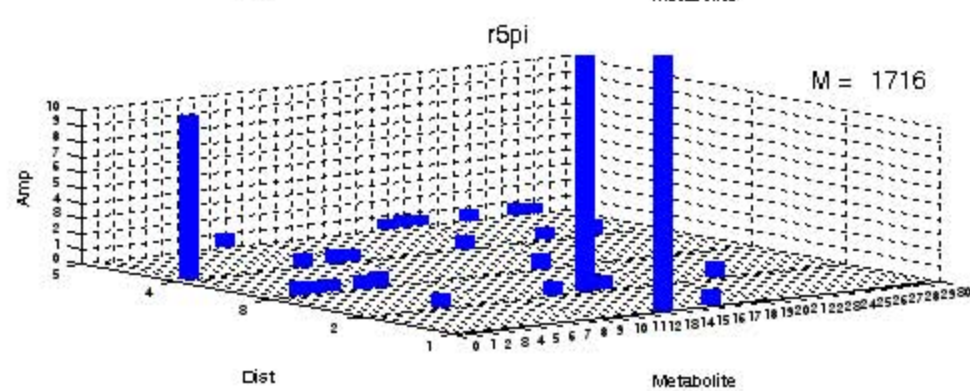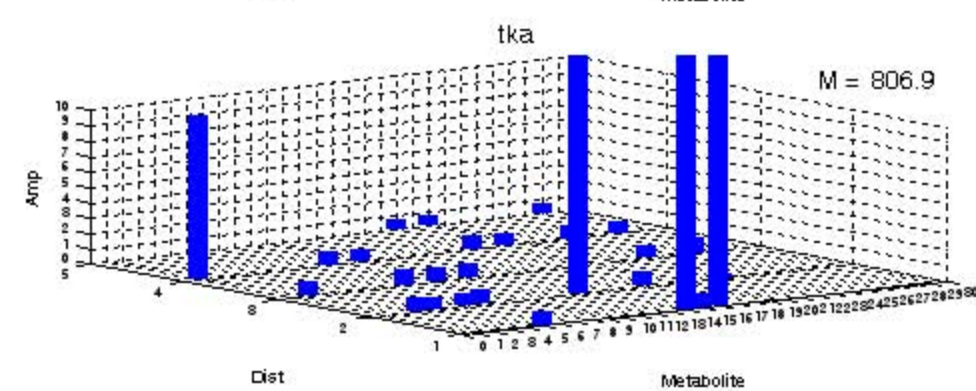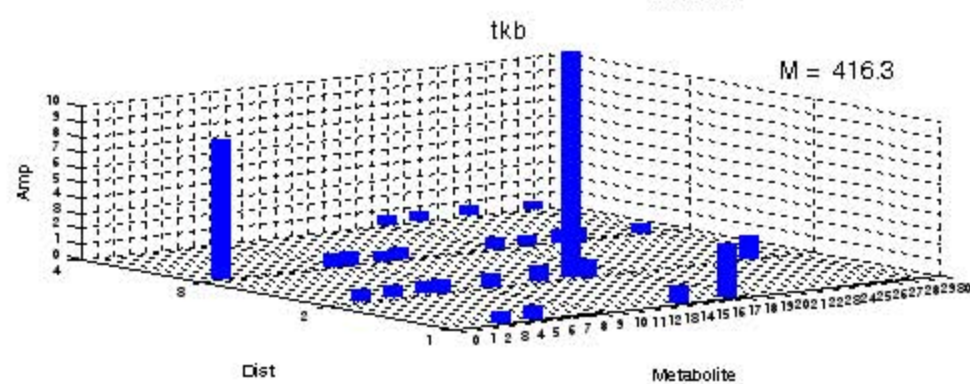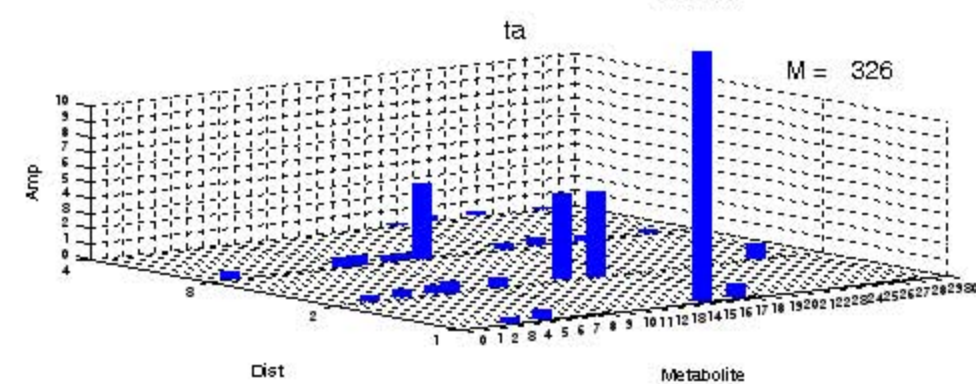

Supplement: Additional file 2 — Impacts on system states in the metabolite-centric view. The file is in the format of *.pdf, with each plot showing the impacts of enzymes deletions on the metabolite kinetics. The metabolite indexes, impact distances and state uation amplitudes form the 3 dimensions. Legends are the same as those in Figure 2. [file 1752-0509-6-S1-S11-S2.pdf]

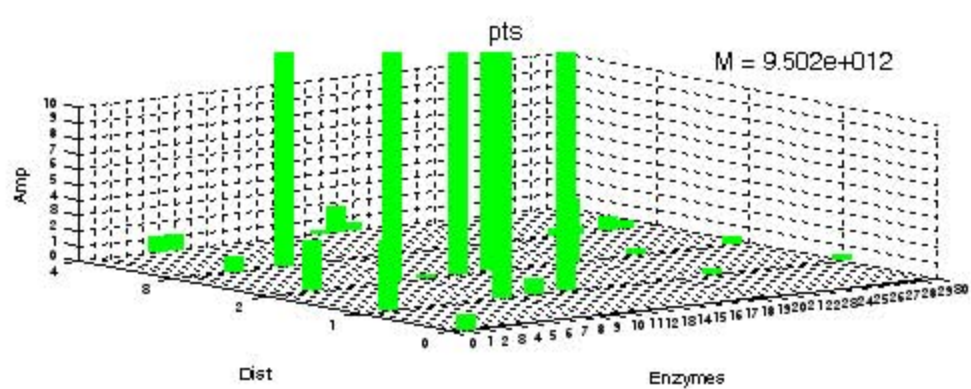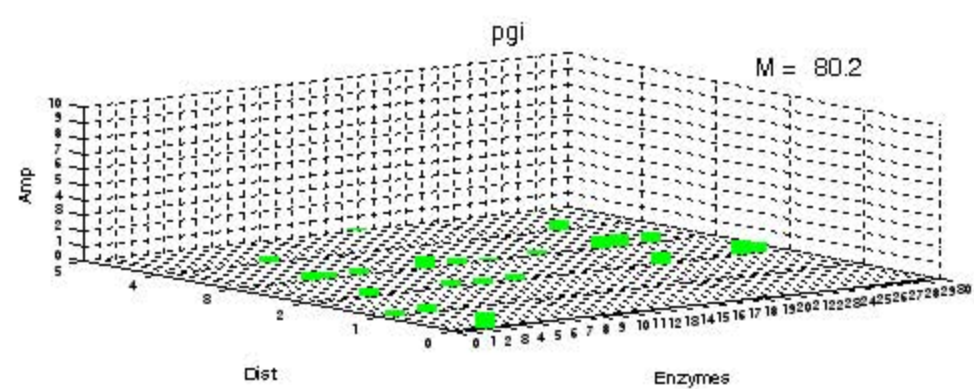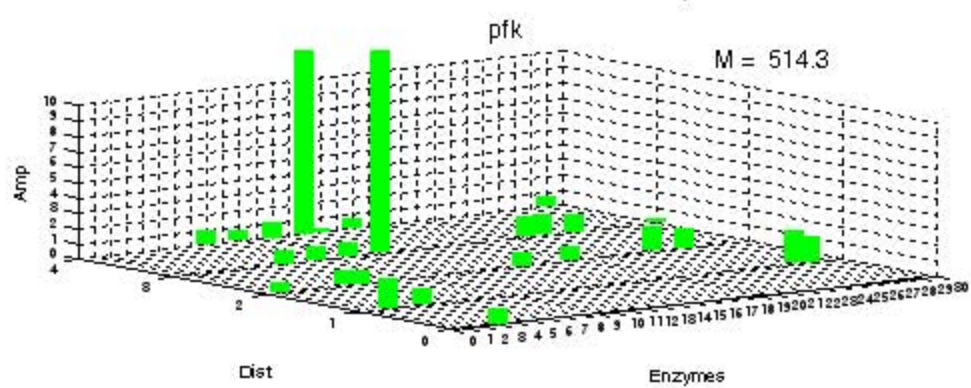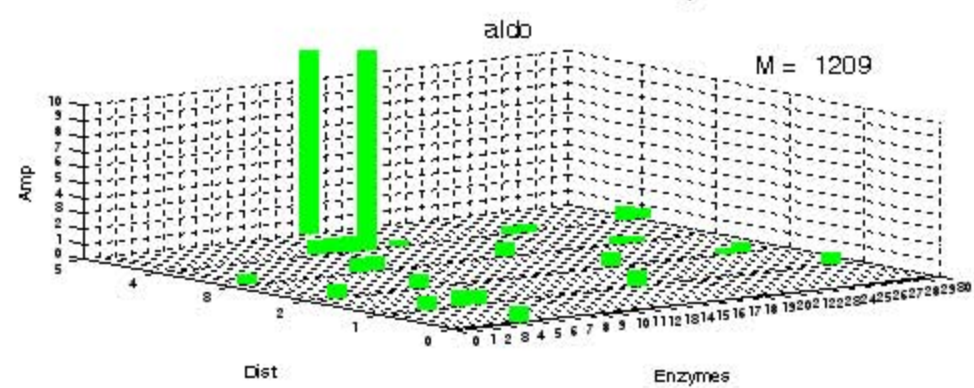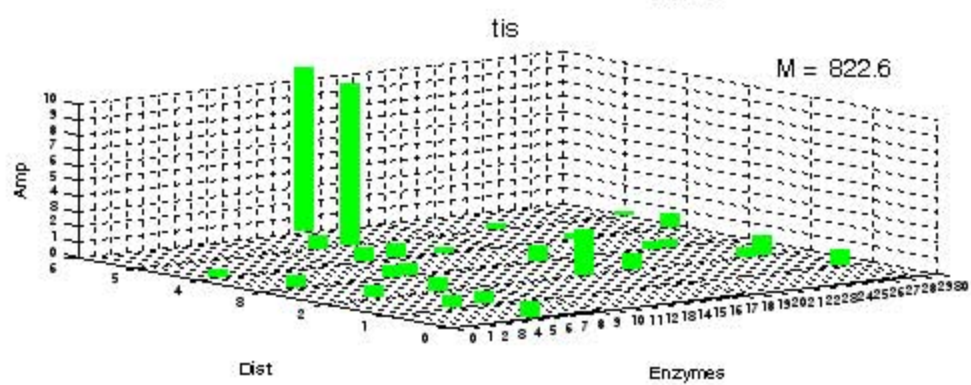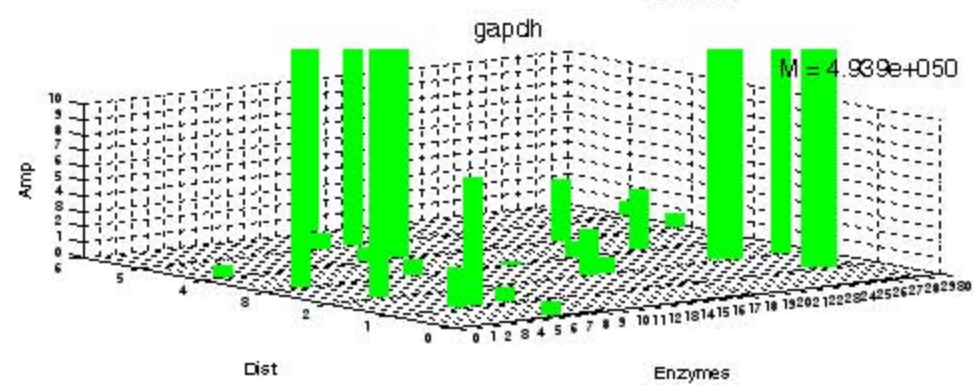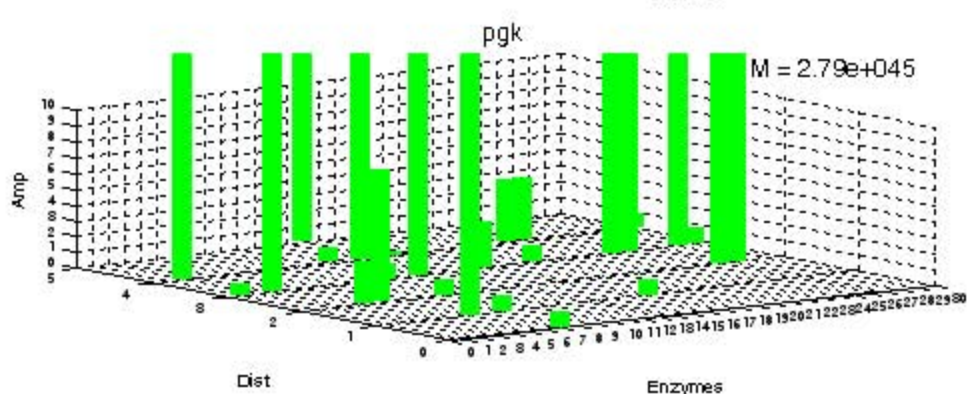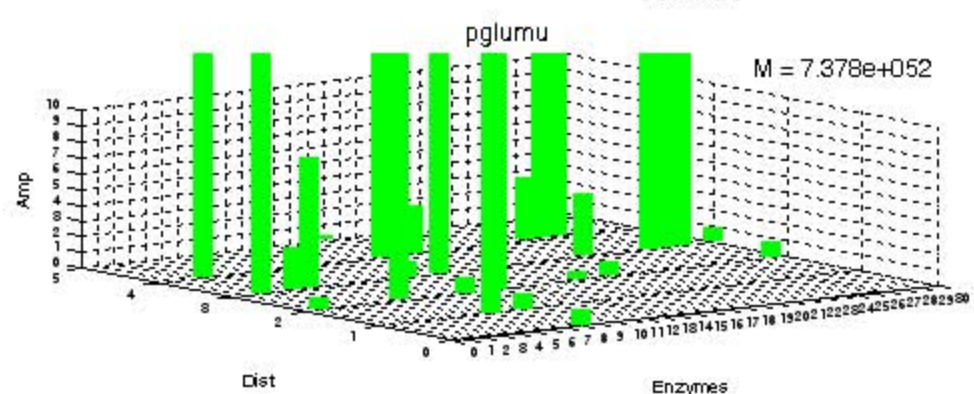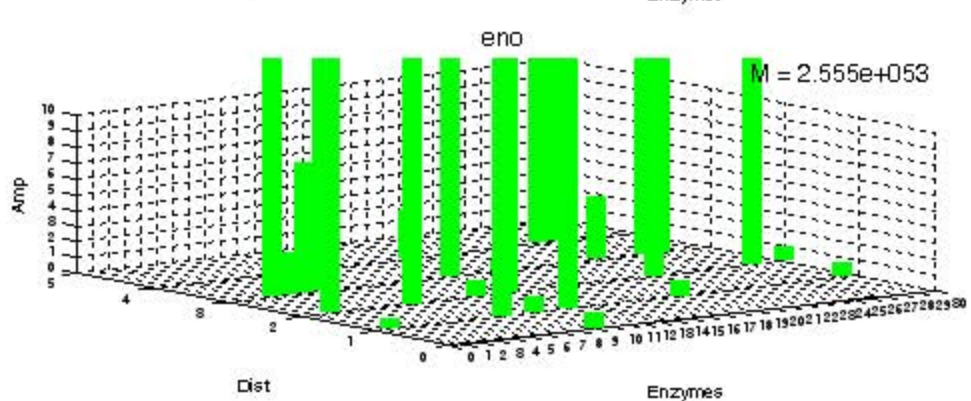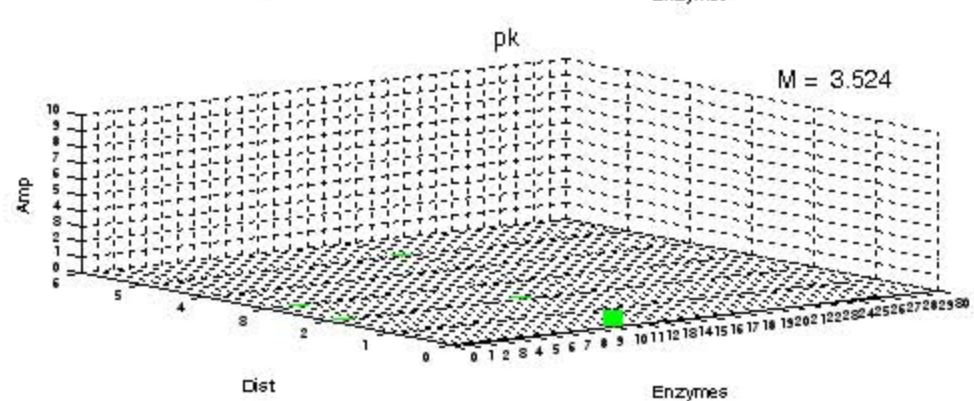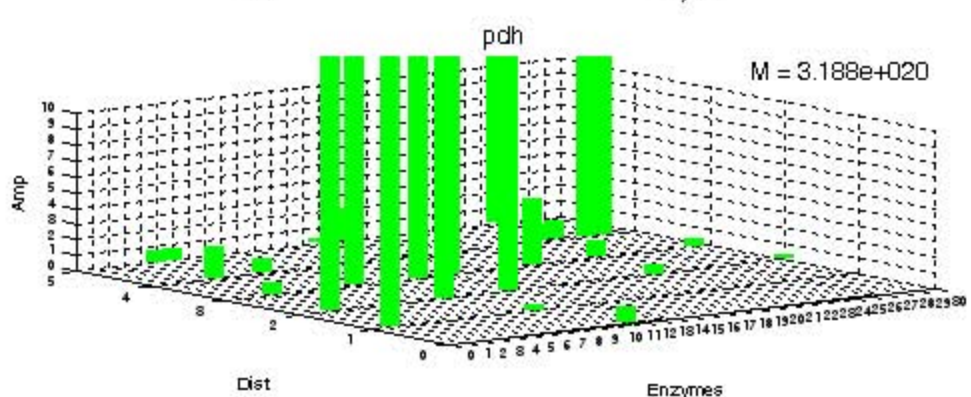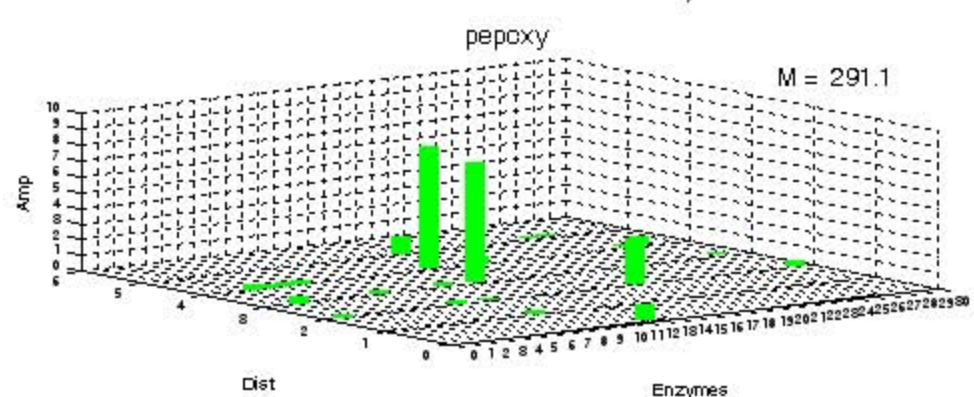

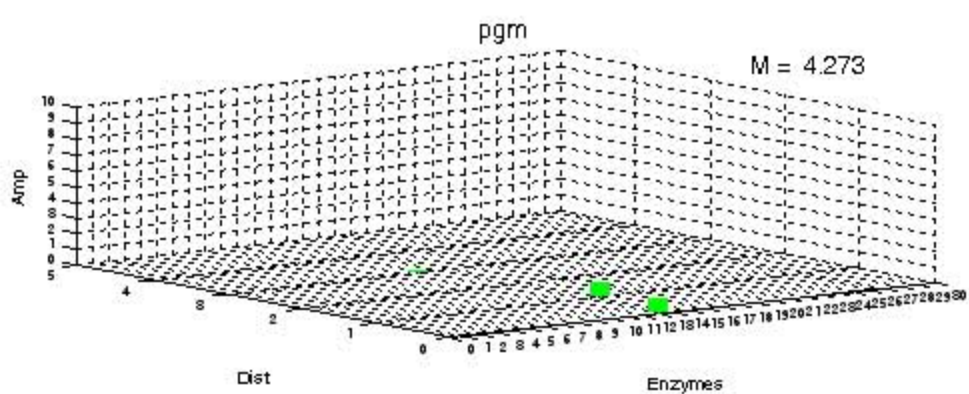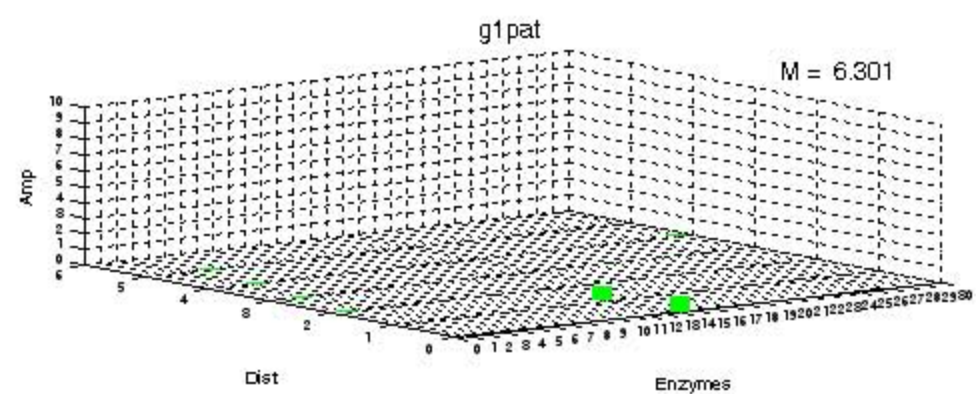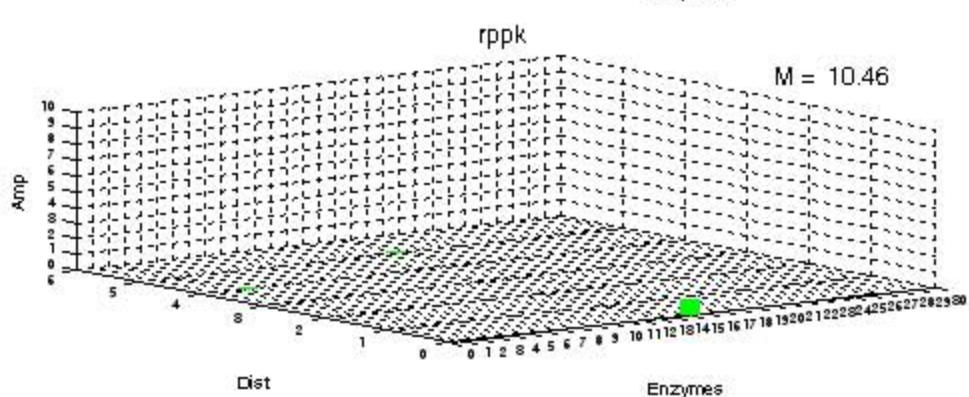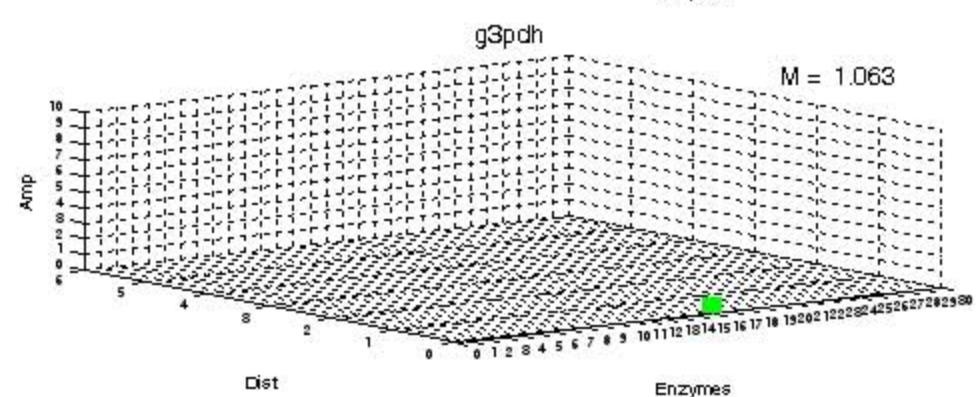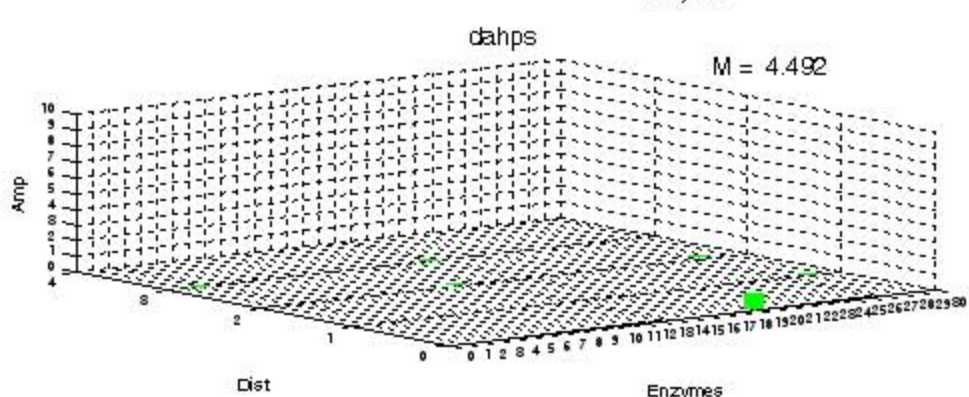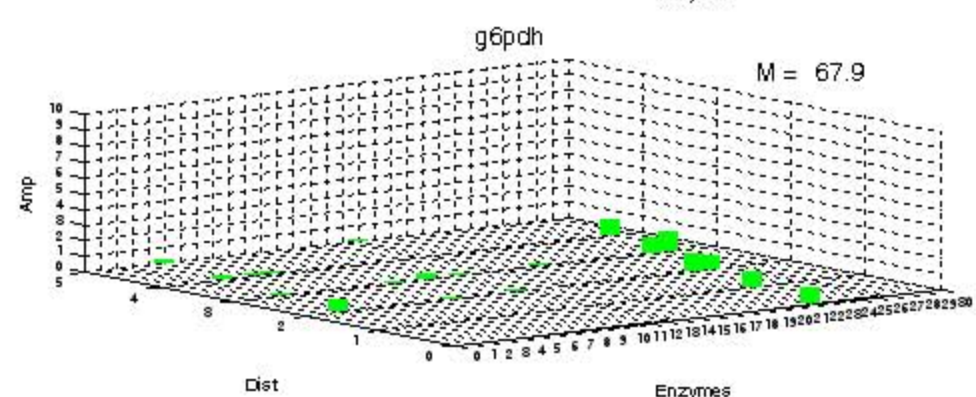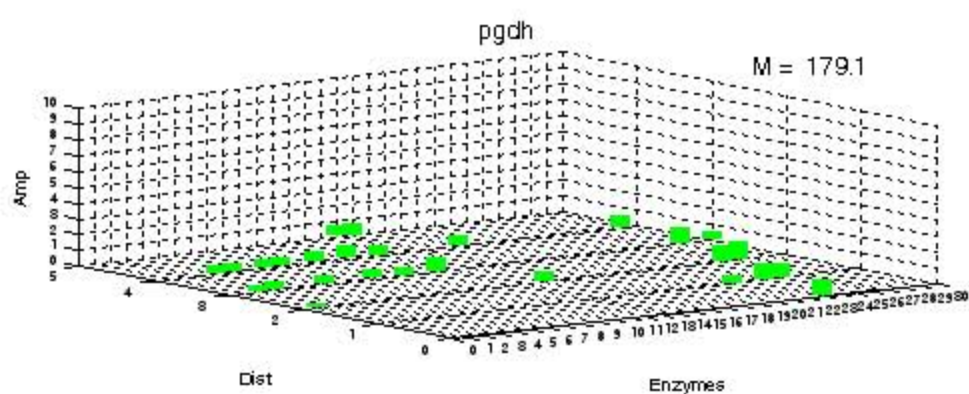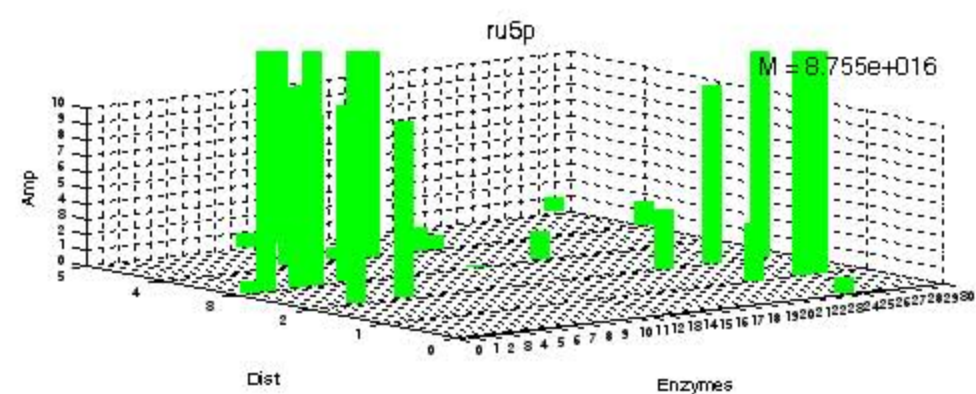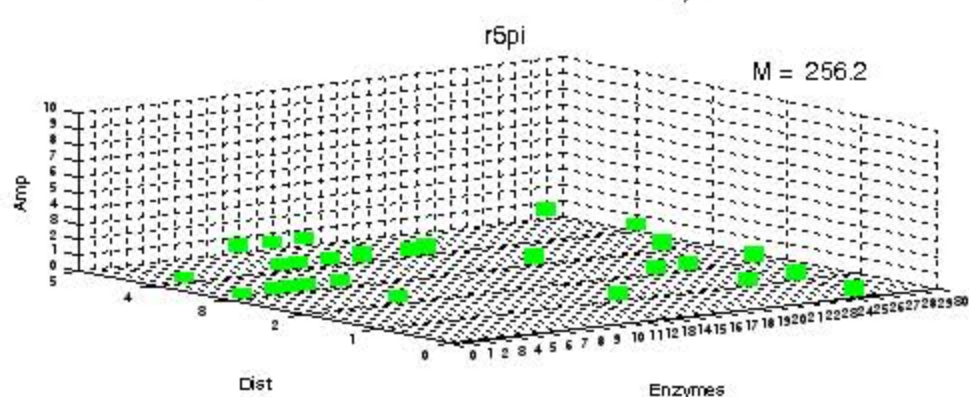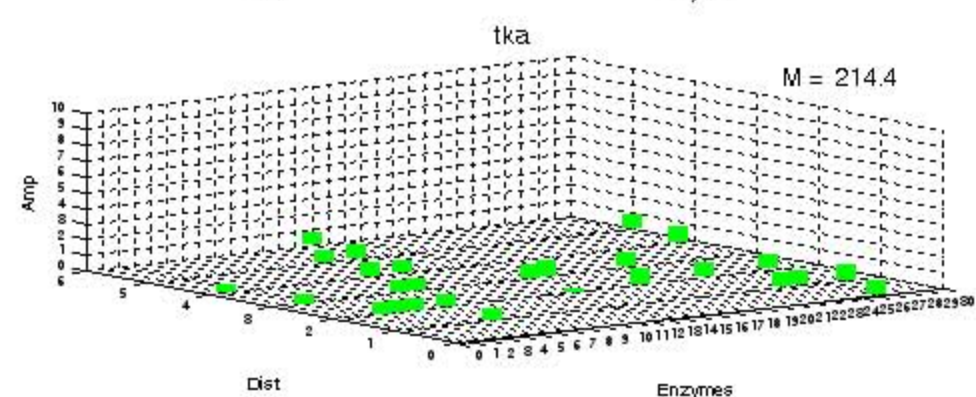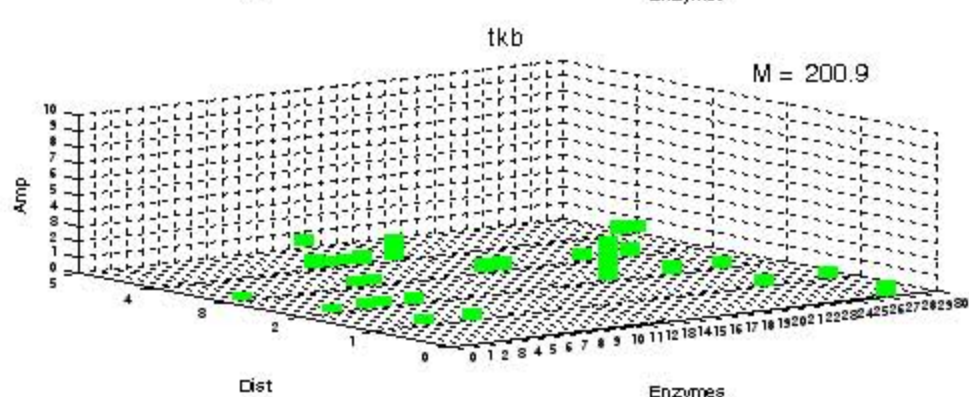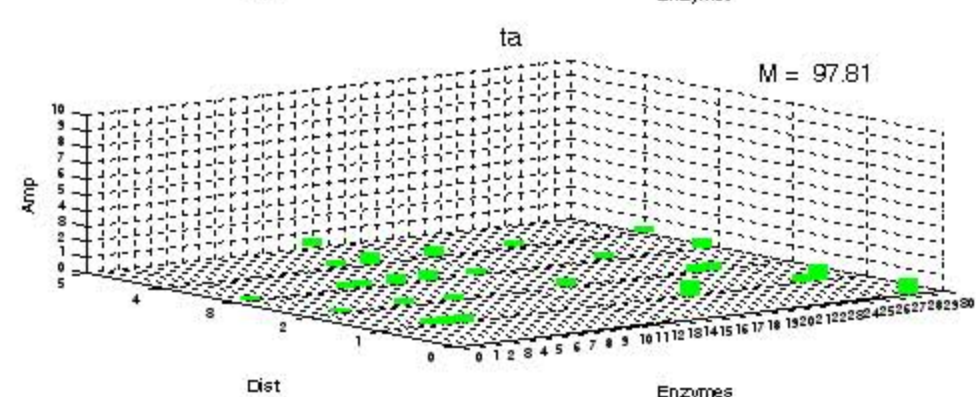

Supplement: Additional file 3 — Impacts on system states in the enzyme-centric view. The file is in the format of *.pdf, with each plot showing the impacts of enzymes deletions on the kinetic fluxes. The reaction/enzyme indexes, impact distances and flux fluctuation amplitudes form the 3 dimensions. Legends are the same as those in Additional file 2. [file 1752-0509-6-S1-S11-S3.pdf]

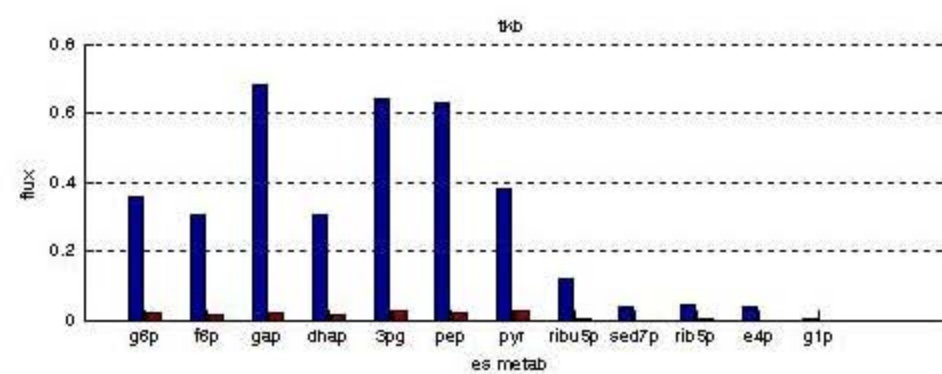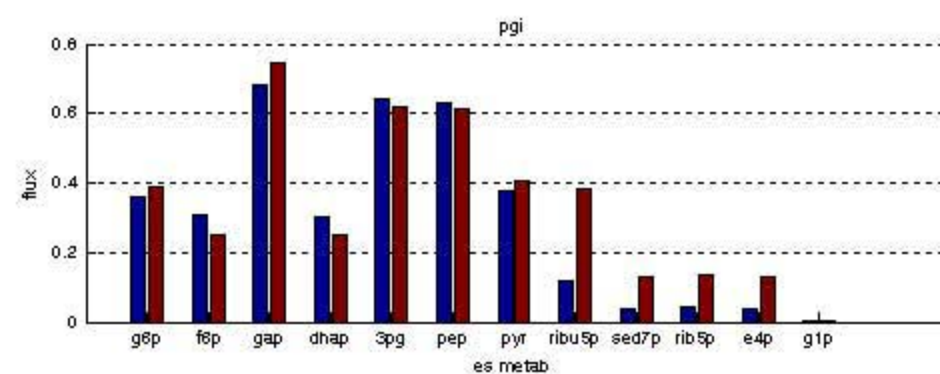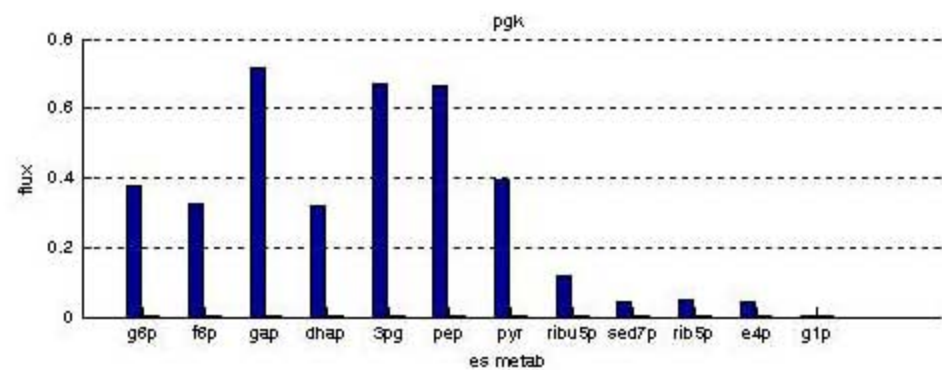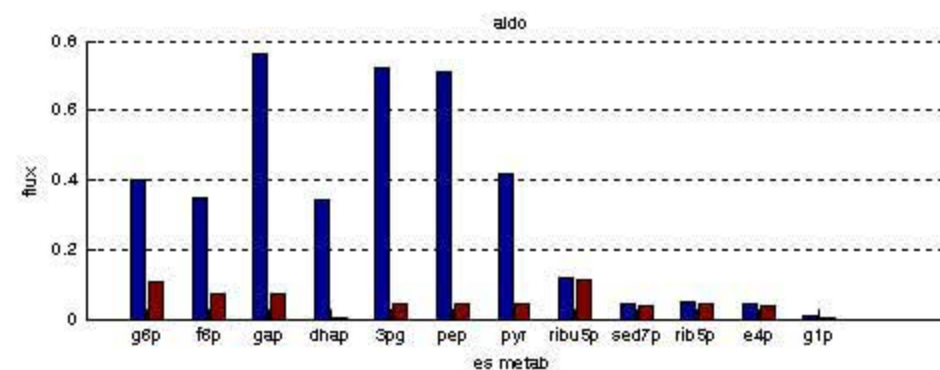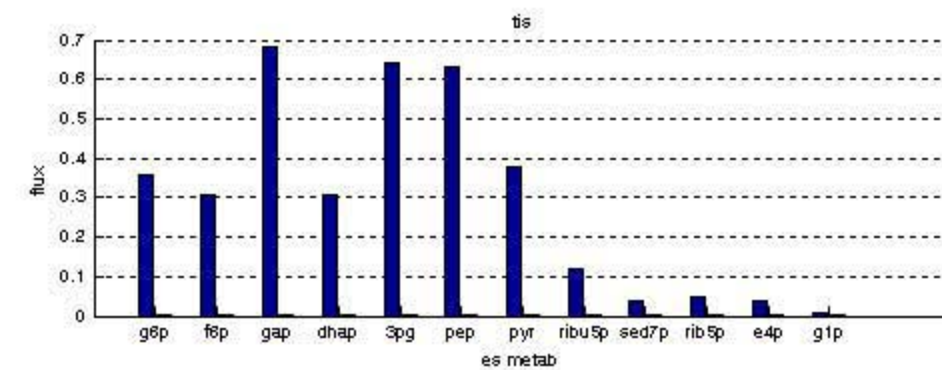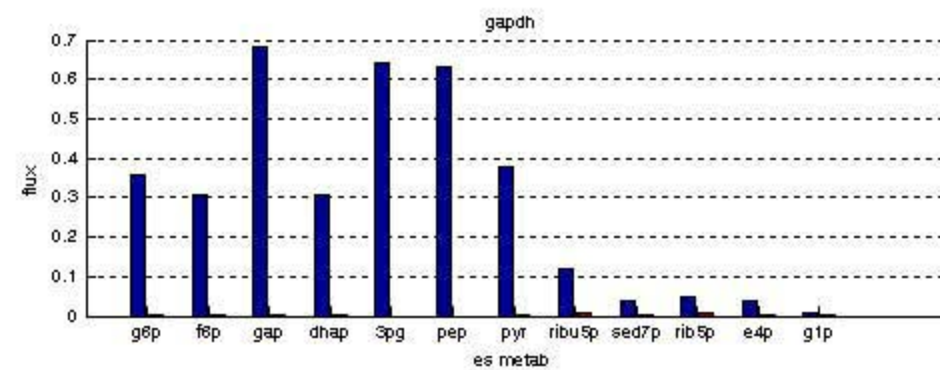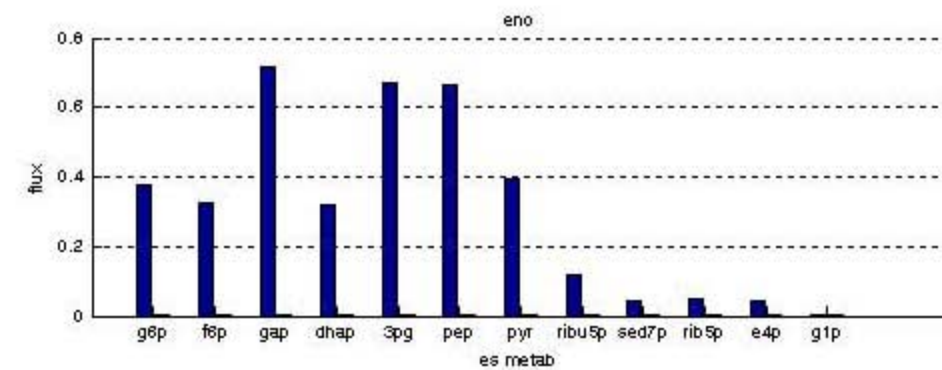

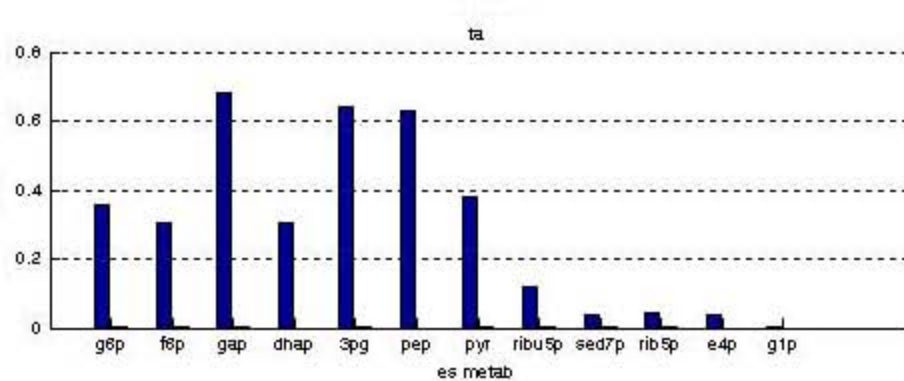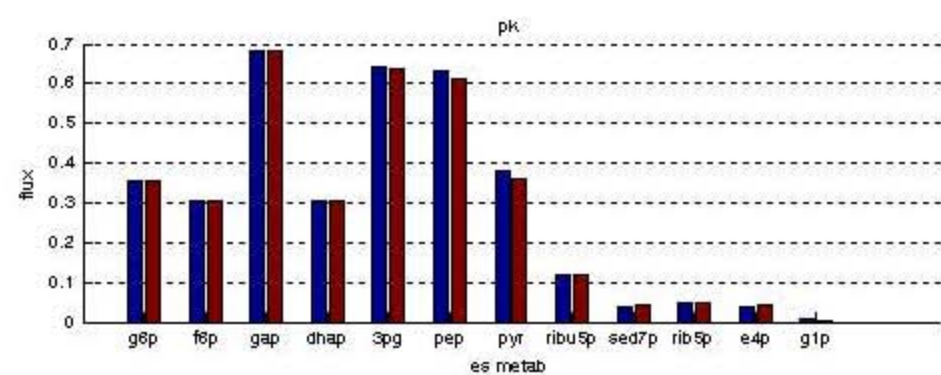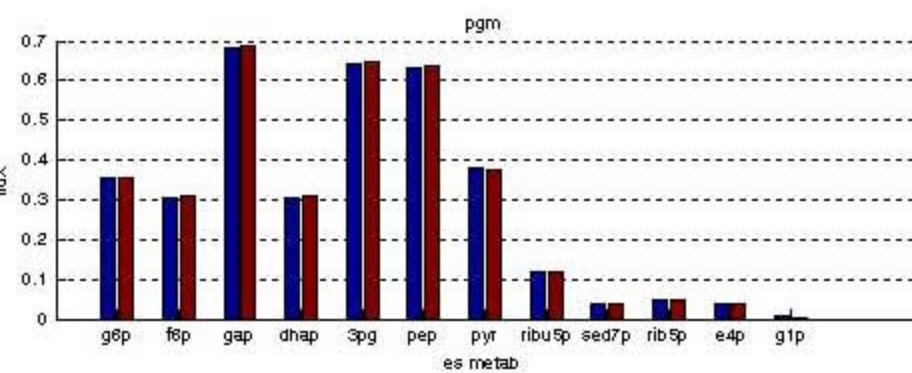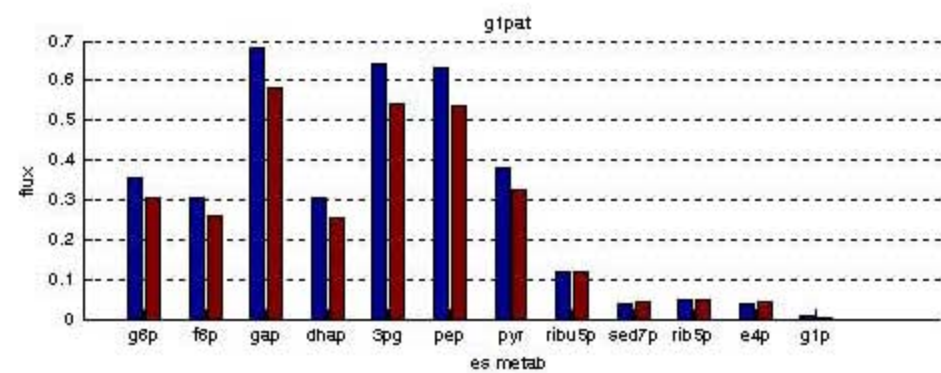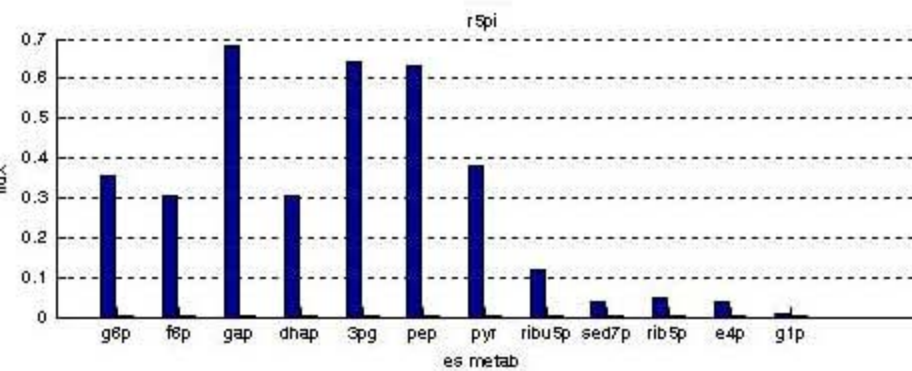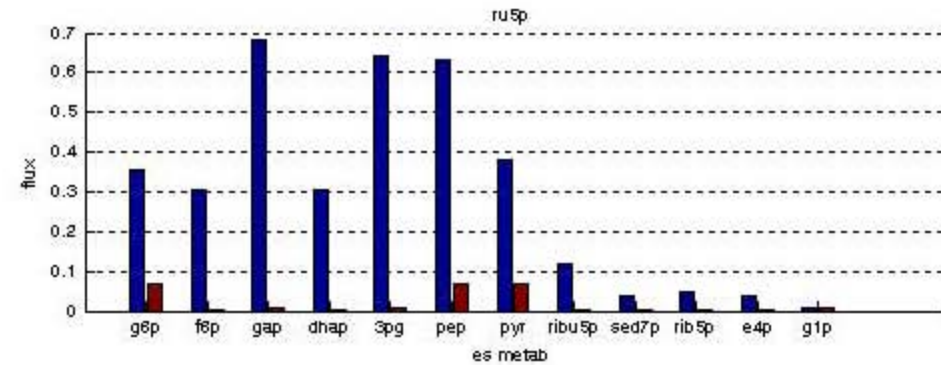

Supplement: Additional file 5 — The flux-sum validations of critical and uncritical enzymes. The file is in the format of *.pdf, with each plot showing the flux-sums of the essential metabolites before and after the deletion of an enzyme. Deletions of all presented enzymes are shown. The metabolite symbols and flux-sum values form the lateral and vertical dimensions. Legends are the same as those in Figure 5. [file 1752-0509-6-S1-S11-S5.pdf]

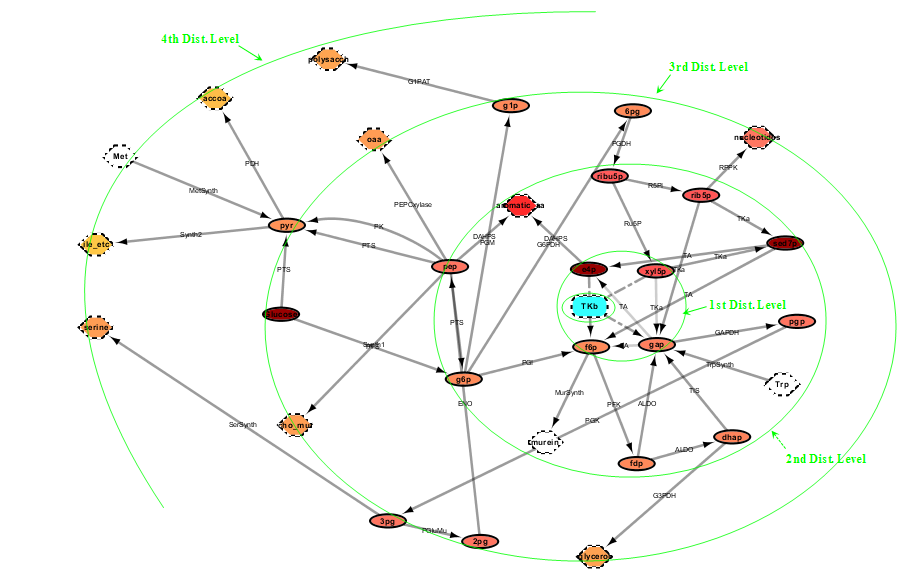

Supplement: Additional file 7 — High resolution images of Figure 6A and 6B. The two files (in the format of *.png) are the high resolution versions of Figure 6A and 6B, respectively. [file 1752-0509-6-S1-S11-S7.png]

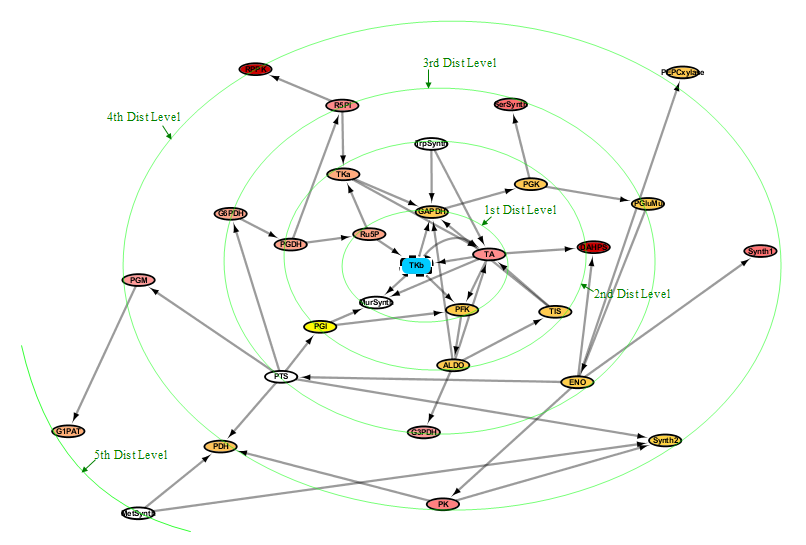

Supplement: Additional file 8 — High resolution images of Figure 6A and 6B. The two files (in the format of *.png) are the high resolution versions of Figure 6A and 6B, respectively. [file 1752-0509-6-S1-S11-S8.png]
