# Supplementary material for: Characterizing criticality of proteins by systems dynamics: Escherichia coli central carbon metabolism as a working example
Source: BMC Syst Biol. 2012 Jul 16;6(Suppl 1):S11. doi: 10.1186/1752-0509-6-S1-S11 (PMC3402961; doi:10.1186/1752-0509-6-S1-S11)
Supplement: Additional file 4 — The catalogue of the results of dynamical stability analysis. The file categorizes all enzymes that generate topologically equivalent system orbit structures, when deleted. Enzymes in the same category exert similar impacts on the qualitative dynamics of the system. The file is in the format of MS Word electronic table (*.doc). [file 1752-0509-6-S1-S11-S4.doc]

Additional file 4

| Category | Enzymes |
| --- | --- |
| 1 | PGI, PK, PEPCxylase, PGM, G1PAT, RPPK, G6PDH, G3PDH, DAHPS |
| 2 | TKb, TA |
| 3 | PGDH, TKa, R5PI |
| 4 | ALDO, PFK, TIS |
| 5 | PGK, GAPDH, PDH, PTS, PGluMu, ENO, Ru5P |

Notes: Deletions of enzymes in the same category result in similar orbit structures; while the orbit structures are qualitatively different when deleting enzymes belonging to different categories.
