# Supplementary material for: Characterizing criticality of proteins by systems dynamics: Escherichia coli central carbon metabolism as a working example
Source: BMC Syst Biol. 2012 Jul 16;6(Suppl 1):S11. doi: 10.1186/1752-0509-6-S1-S11 (PMC3402961; doi:10.1186/1752-0509-6-S1-S11)
Supplement: Additional file 6 — Description of the modeling. The file is the supplementary text of the modeling. It is in the format of *.doc and includes detailed descriptions of metabolites, enzymes, kinetic rate equations and ODEs. [file 1752-0509-6-S1-S11-S6.doc]

Additional file 6

*1. Metabolites*

| Index | Abbreviation | Metabolite |
| --- | --- | --- |
| 1 | glc | glucose |
| 2 | g6p | glucose-6-phosphate |
| 3 | f6p | fructose-6-phosphate |
| 4 | fdp | fructose-1,6-biphosphate |
| 5 | gap | glyceraldehydes-3-phosphate |
| 6 | dhap | dihydroxyacetonephosphate |
| 7 | pgp | 1,3-diphosphoglycerate |
| 8 | 3pg | 3-phosphoglycerate |
| 9 | 2pg | 2-phosphoglycerate |
| 10 | pep | phosphoenolpyruvate |
| 11 | pyr | pyruvate |
| 12 | 6pg | 6-phosphogluconate |
| 13 | ribu5p | ribulose-5-phosphate |
| 14 | xyl5p | xylulose-5-phosphate |
| 15 | sed7p | sedoheptulose-7-phosphate |
| 16 | rib5p | ribose-5-phosphate |
| 17 | e4p | erythrose-4-phosphate |
| 18 | g1p | glucose-1-phosphate |
| 19 | accoa | acetyl-coenzyme A |
| 20 | oaa | oxaloacetate |
| 21 | polysacch | polysaccharide |
| 22 | nuclt | nucleotides |
| 23 | gly | glycerol/glycerol-3-phosphate |
| 24 | ser | serine |
| 25 | mur | murine |
| 26 | aro_aa | aromatic amino acid |
| 27 | cho | chorismate |
| 28 | ile_lala_kival_dipim | isoleucine, alanine, ketoisovalerate, diaminopimelate |
| 29 | met | methionine |
| 30 | trp | tryptophan |

*2. Enzymes/Reactions*

| Index | Abbreviation | Enzyme |
| --- | --- | --- |
| 1 | PTS | phosphotransferase system |
| 2 | PGI | glucose-6-phosphate isomerase |
| 3 | PFK | phosphofructokinase |
| 4 | ALDO | aldolase |
| 5 | TIS | triosephosphate isomerase |
| 6 | GAPDH | glyceraldehydes-3-phosphate dehydrogenase |
| 7 | PGK | phosphoglycerate kinase |
| 8 | PGluMu | phosphoglycerate mutase |
| 9 | ENO | enolase |
| 10 | PK | pyruvate kinase |
| 11 | PDH | pyruvate dehydrogenase |
| 12 | PEPCxylase | phosphoenolpyruvate carboxylase |
| 13 | PGM | phosphoglucomutase |
| 14 | G1PAT | glucose-1-phosphate adenyltransferase |
| 15 | RPPK | ribose-phosphate pyrophosphokinase |
| 16 | G3PDH | glycerol-3-phosphate dehydrogenase |
| 17 | SerSynth | serine synthesis |
| 18 | MurSynth | murine synthesis |
| 19 | DAHPS | 3-deoxy-7-phosphoheptulonate synthase |
| 20 | TrpSynth | tryptophan synthesis |
| 21 | MetSynth | methionine synthesis |
| 22 | G6PDH | glucose-6-phosphate dehydrogenase |
| 23 | PGDH | 6-phosphogluconate dehydrogenase |
| 24 | Ru5P | ribulose-5-phosphate epimerase |
| 25 | R5PI | ribose-5-phosphate isomerase |
| 26 | TKa | transketolase-a |
| 27 | TKb | transketolase-b |
| 28 | TA | transaldolase |
| 29 | Synth1 | synthesis of chorismate, murein |
| 30 | Synth2 | synthesis of isoleucine, alanine, ketoisovalerate, diaminopimelate |

*3. Reaction Rate Equations*

PTS:

PGI:

PFK:

ALDO:

TIS:

GAPDH:

PGK:

PGluMu:

ENO:

PK:

PDH:

$$r_{PDH}=\frac{r_{PDH}^{\max}[pyr]^{n_{4}}}{K_{PDH,pyr}+[pyr]^{n_{4}}}$$

PEPCxylase:

PGM:

G1PAT:

RPPK:

$$r_{RPPK}=\frac{r_{RPPK}^{\max}[rib5p]}{K_{RPPK,rib5p}+[rib5p]}$$

G3PDH:

$$r_{G3PDH}=\frac{r_{G3PDH}^{\max}[dhap]}{K_{G3PDH,dhap}+[dhap]}$$

SerSynth:

$$r_{SerSynth}=\frac{r_{SerSynth}^{\max}[3pg]}{K_{SerSynth,3pg}+[3pg]}$$

MurSynth:

$$r_{MurSynth}=r_{MurSynth}^{\max}$$

DAHPS:

$$r_{DAHPS}=\frac{r_{DAHPS}^{\max}[e4p]^{n_{7}}[pep]^{n_{8}}}{\left(K_{DAHPS,e4p}+[e4p]^{n_{7}}\right)\left(K_{DAHPS,pep}+[pep]^{n_{8}}\right)}$$

TrpSynth:

$$r_{TrpSynth}=r_{TrpSynth}^{\max}$$

MetSynth:

$$r_{MetSynth}=r_{MetSynth}^{\max}$$

G6PDH:

PGDH:

Ru5P:

R5PI:

TKa:

TKb:

TA:

Synth1:

$$r_{Synth1}=\frac{r_{Synth1}^{\max}[pep]}{K_{Synth1,pep}+[pep]}$$

Synth2:

$$r_{Synth2}=\frac{r_{Synth2}^{\max}[pyr]}{K_{Synth2,pyr}+[pyr]}$$

Notes: Kinetic parameters were taken from literature [1-6]. The kinetic equations took into consideration for the reversible reactions using equilibrium constants *K_eq_* as in Eqn 2, 4 – 9, 13, and 24 – 28. Because of the specificity of enzymes, reverse effects were regarded as spontaneous chemical reactions dominated by *K_eq_*. By modifying the equations with the ratio of the products and *K_eq_*, the net effects of the forward enzymatic reactions were approximated.

*4. Cometabolites*

$$[atp]=4.27-4.163\frac{t}{0.657+1.43t+0.364t^{2}}$$

$$[adp]=0.582+1.73\left(2.731^{-0.15t}\right)\left(0.12t+0.000214t^{3}\right)$$

$$[amp]=0.123+7.25\frac{t}{7.25+1.47t+0.17t^{2}}+1.073\frac{t}{1.29+8.05t}$$

$$[nadph]=0.062+0.332\left(2.718^{-0.464t}\right)\left(0.0166t^{1.58}+0.000166t^{4.73}+1.13\times10^{-10}t^{7.89}+1.36\times10^{-13}t^{11.0}+1.23\times10^{-16}t^{14.2}\right)$$

$$[nadp]=0.159+0.00554\frac{t}{2.8+0.271t+0.01t^{2}}+0.182\frac{t}{4.81+0.526t}$$

$$[nadh]=0.0934+0.0011\left(2.371^{-0.123t}\right)\left(0.844t+0.104t^{3}\right)$$

$$[nad]=1.314+1.314\left(2.73^{-0.0435t-0.342}\right)-\frac{\left(t+7.871\right)\left(2.73^{-0.0218t-0.171}\right)}{8.481+t}$$

Notes: In reaction rate equations, there were quantities of cometabolites ATP, ADP, AMP, NADPH, NADP, NADH, and NAD. They were fitted experimentally as in Eqn 31 – 37 [1]. However, they could be regarded as constants in the system as these equations all had limits. Moreover, when the time variable exceeded 100 time unit, the values of the 7 equations almost equaled to their respectively limits. Since equilibriums were properties with respect to adequately long time intervals, therefore we substituted the 7 cometabolites with their respective constant limit values in the analysis of system equilibrium and stability, so as to make the system autonomous.

*5. Ordinary Differential Equations (ODE)*

Internal metabolites:

$$\frac{d[glc]}{dt}=D\left(C_{glc,feed}-[glc]\right)-\frac{C_{x}}{\rho_{x}}r_{PTS}$$

$$\frac{d[g6p]}{dt}=r_{PTS}-r_{PGI}-r_{G6PDH}-r_{PGM}-\mu[g6p]$$

$$\frac{d[f6p]}{dt}=r_{PGI}-r_{PFK}+r_{TKb}+r_{TA}-2r_{MurSynth}-\mu[f6p]$$

$$\frac{d[fdp]}{dt}=r_{PFK}-r_{ALDO}-\mu[fdp]$$

$$\frac{d[gap]}{dt}=r_{ALDO}+r_{TIS}-r_{GAPDH}+r_{TKa}+r_{TKb}-r_{TA}+r_{TrpSynth}-\mu[gap]$$

$$\frac{d[dhap]}{dt}=r_{ALDO}-r_{TIS}-r_{G3PDH}-\mu[dhap]$$

$$\frac{d[pgp]}{dt}=r_{GAPDH}-r_{PGK}-\mu[pgp]$$

$$\frac{d[3pg]}{dt}=r_{PGK}-r_{PGluMu}-r_{SerSynth}-\mu[3pg]$$

$$\frac{d[2pg]}{dt}=r_{PGluMu}-r_{ENO}-\mu[2pg]$$

$$\frac{d[pep]}{dt}=r_{ENO}-r_{PK}-r_{PTS}-r_{PEPCxylase}-r_{DAHPS}-r_{Synth1}-\mu[pep]$$

$$\frac{d[pyr]}{dt}=r_{PK}+r_{PTS}-r_{PDH}+r_{MetSynth}-r_{Synth2}-\mu[pyr]$$

$$\frac{d[6pg]}{dt}=r_{G6PDH}-r_{PGDH}-\mu[6pg]$$

$$\frac{d[ribu5p]}{dt}=r_{PGDH}-r_{Ru5P}-r_{R5PI}-\mu[ribu5p]$$

$$\frac{d[xyl5p]}{dt}=r_{Ru5P}-r_{TKa}-r_{TKb}-\mu[xyl5p]$$

$$\frac{d[sed7p]}{dt}=r_{TKa}-r_{TA}-\mu[sed7p]$$

$$\frac{d[rib5p]}{dt}=r_{R5PI}-r_{TKa}-r_{RPPK}-\mu[rib5p]$$

$$\frac{d[e4p]}{dt}=r_{TA}-r_{TKb}-r_{DAHPS}-\mu[e4p]$$

$$\frac{d[g1p]}{dt}=r_{PGM}-r_{G1PAT}-\mu[g1p]$$

Notes: Parameters *D, C_glc,feed_, C_x_, ρ_x_* and *μ* were taken from literature [1].

External metabolites:

$$\frac{d[accoa]^{*}}{dt}=r_{PDH}$$

$$\frac{d[oaa]^{*}}{dt}=r_{PEPCxylase}$$

$$\frac{d[polysacch]^{*}}{dt}=r_{G1PAT}$$

$$\frac{d[nuclt]^{*}}{dt}=r_{RPPK}$$

$$\frac{d[gly]^{*}}{dt}=r_{G3PDH}$$

$$\frac{d[ser]^{*}}{dt}=r_{SerSynth}$$

$$\frac{d[mur]^{*}}{dt}=r_{MurSynth}+r_{Synth1}$$

$$\frac{d[aro\_aa]^{*}}{dt}=r_{DAHPS}$$

$$\frac{d[cho]^{*}}{dt}=r_{Synth1}$$

$$\frac{d[ile\_lala\_kival\_dipim]^{*}}{dt}=r_{Synth2}$$

$$\frac{d[trp]^{*}}{dt}=0$$

$$\frac{d[met]^{*}}{dt}=0$$

Notes: Eqn 56 – 67 did not stand for mass-balance of the external metabolites. They just represented the state changes of external metabolites due to the respective reactions through which the system connected to them (i.e. the single-side actions exerted by the system on external metabolites).

*6. The Metabolic System Model*

We wrote the ODEs in the matrix form, by setting:

$$\begin{matrix} X=\left(glc,g6p,\ldots,g1p\right)^{T} \\ R=\left(r_{PTS},r_{PGI},\ldots,r_{Synth2}\right)^{T} \end{matrix}$$

$$B=\left[\begin{matrix} -D & & & \\ & -\mu& & \\ & & \ddots& \\ & & & -\mu\end{matrix}\right]\cdot X+\left[\begin{matrix} DC_{glc,feed} \\ 0 \\ \vdots\\ 0 \end{matrix}\right]$$

we wrote Eqn 38 – 55 as the following,

$$\frac{dX}{dt}=A\cdot R(X,P)+B(X,P)$$

which is exactly in the same form of Eqn 1 as in the main text. We regarded Eqn 68 as the metabolic system model and used it for analysis throughout the present work. Eqn 56 – 67 are regarded as output equations which were estimated from the solution results of Eqn 68, but not solved directly. Eqn 56 – 67 were added in the analysis of system state fluctuation because it was important to assess how the influences would propagate to external bio-processes (single-side effects on external metabolites by the system); but they could not be added in the analysis of system equilibrium and stability because the external metabolites themselves were not subjected to mass-balance within our system model (they connected to other pathways beyond our system).

*7. References*

1. Chassagnole C, Noisommit-Rizzi N, Schmid JW, Mauch K, Reuss M: **Dynamic modeling of the central carbon metabolism of *Escherichia coli***. *Biotechnol Bioeng* 2002, **79**:53-73.

2. Theobald U, Mailinger W, Baltes M, Rizzi M, Reuss M: **In vivo analysis of metabolic dynamics in *Saccharomyces cerevisiae*: I. Experimental observations**. *Biotechnol Bioeng* 1997, **55**:305-316.

3. Rizzi M, Baltes M, Theobald U, Reuss M: **In vivo analysis of metabolic dynamics in *Saccharomyces cerevisiae*: II. Mathematical model**. *Biotechnol Bioeng* 1997, **55**:592-608.

4. Duggleby RG: **Product inhibition of reversible enzyme-catalysed reactions**. *Biochim Biophys Acta* 1994, **1209**:238-240.

5. Bakker BM, Michels PAM, Opperdoes FR, Westerhoff HV: **Glycolysis in bloodstream from Trypanosoma brucei can be understood in terms of the kinetics of the glycolytic enzymes**. *J Biol Chem* 1997, **272**:3207-3215.

6. Vaseghi S, Baumeister A, Rizzi M, Reuss M: **In vivo dynamics of the pentose phosphate pathway in *Saccharomyces cerevisiae***. *Metab Eng* 1999, **1**:128-140.
